# Comprehensive and updated review on anti-oxidant effects of *Nigella sativa* and its constituent, thymoquinone, in various disorders

**DOI:** 10.22038/IJBMS.2024.75985.16453

**Published:** 2024

**Authors:** Mohammad Reza Aslani, Saeideh Saadat, Mohammad Hossein Boskabady

**Affiliations:** 1 Applied Biomedical Research Center, Mashhad University of Medical Sciences, Mashhad, Iran; 2 Lung Inflammatory Diseases Research Center, Faculty of Medicine, Ardabil University of Medical Sciences, Ardabil, Iran; 3 Department of Physiology, School of Medicine, Zahedan University of Medical Sciences, Zahedan, Iran; 4 Department of Physiology, Faculty of Medicine, Mashhad University of Medical Sciences, Mashhad, Iran

**Keywords:** Anti-oxidants, Essential oil, Plant extracts, Nigella sativa, Oxidative stress, Thymoquinone

## Abstract

Several pharmacological effects were described for *Nigella sativa *(*N. sativa*) seed and it has been used traditionally to treat various diseases. In this review article, the updated and comprehensive anti-oxidant effects of *N. sativa* and its main constituent, thymoquinone (TQ), on various disorders are described. The relevant articles were retrieved through PubMed, Science Direct, and Scopus up to December 31, 2023. Various extracts and essential oils of *N. sativa* showed anti-oxidant effects on cardiovascular, endocrine, gastrointestinal and liver, neurologic, respiratory, and urogenital diseases by decreasing and increasing various oxidant and anti-oxidant marketers, respectively. The main constituent of the plant, TQ, also showed similar anti-oxidant effects as the plant itself. The anti-oxidant effects of different extracts and essential oils of *N. sativa* were demonstrated in various studies which were perhaps due to the main constituent of the plant, TQ. The findings of this review article suggest the possible therapeutic effect of *N. sativa* and TQ in oxidative stress disorders.

## Introduction

Free radicals are oxygen-containing molecules with an uneven number of electrons with unstable atoms that can damage cells (1). An important feature of these radicals is their oxidizing or reducing role by accepting or losing an electron (2). In biological systems, reactive radical derivatives of oxygen are called reactive oxygen species (ROS), and reactive radical derivatives of nitrogen are called reactive nitrogen species (RNS) (3). ROS is composed of various radical and non-radical species including superoxide (O_2_^•−^), hydrogen peroxide (H_2_O_2_), hydroxyl radical (^•^OH), peroxyl radical (HO_2_^•^), alkoxy radical (RO^•^), hydroperoxyl radical (HO^.^_2_), singlet oxygen (^1^O_2_), and ozone (O_3_). RNS also includes nitric oxide (NO), nitrogen dioxide (NO_2_), nitrous acid (HNO_2_), dinitrogen tetroxide (N_2_O_4_), dinitrogen trioxide (N_2_O_3_), peroxynitrite (ONOO^-^), peroxynitrous acid (ONOOH), and alkyl peroxyn (ROONO) (4). Endogenous sources for ROS/RNS include myeloperoxidase (MPO), nicotinamide adenine dinucleotide phosphate (NADPH) oxidase, lipoxygenase (LPO), cytochrome C oxidase, nitric oxidase synthase (NOS), xanthine oxidase (XO), and angiotensin II (AgII) (4). Exogenous sources for ROS/RNS include tobacco, water and air pollution, alcohol, drugs, heavy metals (eg, gentamicin, tacrolimus, cyclosporine, bleomycin, doxorubicin, morphine, diazinon, potassium bromate, ethanol, acrylamide, arsenic, and bisphenol A), radiation, industrial solvents, and cooking (4). 

On the other hand, anti-oxidants are molecules with defense properties against free radical toxicity and include exogenous and endogenous sources. Endogenous anti-oxidants include enzymatic pathways (such as superoxide dismutase (SOD), catalase (CAT), glutathione peroxidase (GSH-Px)) and non-enzymatic pathways (such as albumin, bilirubin, α-tocopherol (vitamin E), uric acid, and β-carotene) (5, 6). Exogenous anti-oxidants include the following: α-tocopherol (vitamin E), phenolic anti-oxidants (resveratrol, phenolic acids, and flavonoids), ascorbic acid (vitamin C), drugs (such as acetylcysteine), selenium, and zinc (7). 

**Figure 1:**
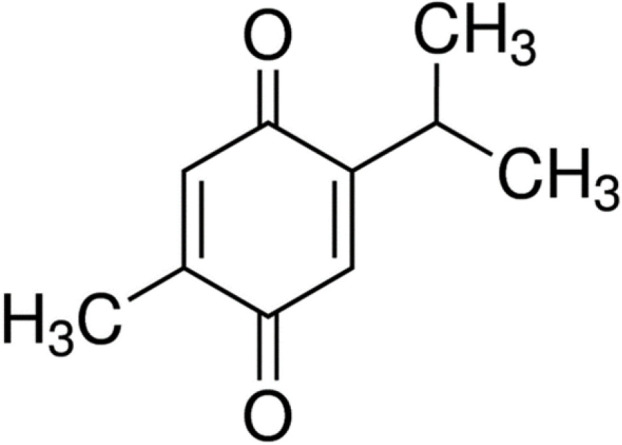
Chemical structure of thymoquinone

Oxidative stress is an imbalance between oxidants and anti-oxidants that can lead to damage to biological systems (1). Controlling specific ROS-mediated signaling pathways by selective targeting agents offers a perspective for a future of more refined redox medicine. This includes enzymatic defense systems such as those controlled by the stress-response transcription factors NRF2 and nuclear factor-κB, the role of trace elements such as selenium, the use of redox drugs, and the modulation of environmental factors is collectively known as the exosome (for example, nutrition, lifestyle, and irradiation) (8). Oxidative stress has been shown to play a key role in the pathogenesis of many diseases such as cardiovascular and pulmonary diseases, cancer, metabolic syndrome, and urogenital and neurological disorders (9). Accordingly, it has been shown that in the treatment of diseases, modulation of oxidative stress responses can be effective in improving disease conditions. Recently, in numerous pieces of evidence, the therapeutic effects of medicinal plants in various diseases have been demonstrated (10-13). One of these medicinal plants is *Nigella sativa* (*N. sativa*) seed and its main active ingredient is thymoquinone (TQ, Figure 1).

*N. sativa* has been used as a food additive plant and has long been used as an herbal medicine in most Southwest Asia, Arabian, African, and Indian countries (13). Numerous studies have shown the therapeutic effects of the plant extracts in various diseases of the nervous system, kidneys, cardiovascular, respiratory, muscular, diabetes and obesity, gastrointestinal, and infertility. In our previous review article (13), the effects of *N. sativa* and its derivatives on respiratory, allergic, and immunologic disorders and its possible mechanisms including their effect on oxidative stress in these disorders were described. However, in the present article, the anti-oxidant effects of the plant and its constituents in several disorders including respiratory diseases are provided in more detail.

There are several reports on the effect of *N. sativa* and its derivatives on oxidative stress (14-20). The effect of *N. sativa*, on biomarkers of oxidative stress was already described (14). The effects of *N. sativa* supplementation on oxidative stress parameters in clinical trials were also reported (15). The protective effects of *N. sativa* in nervous system dysfunctions were also recounted (16). Based on experimental studies, the potential role of *N. sativa* in the management of oxidative stress in several diseases was reported (17). A review study focused on the potential aspects and mechanisms by which TQ acquires its anti-oxidant actions (18). The anti-oxidant aspect of *N. sativa* for its preventive effects on obstructive respiratory diseases was documented (19). The protective effects of *N. sativa* in some diseases such as cancer and diabetes were already reported (20).

However, in this review article, an updated and comprehensive report on the anti-oxidant effects of *N. sativa* and its main constituent, TQ, based on experimental and clinical studies, on respiratory, cardiovascular, gastrointestinal, renal, diabetes, obesity, and reproductive disorders were provided. Although there have been a number of review studies on the anti-oxidant effects of *N. sativa* and its constituents, the current article has presented the results of new studies as well as its anti-oxidant effects in various disorders.

**Method**

**Figure 2:**
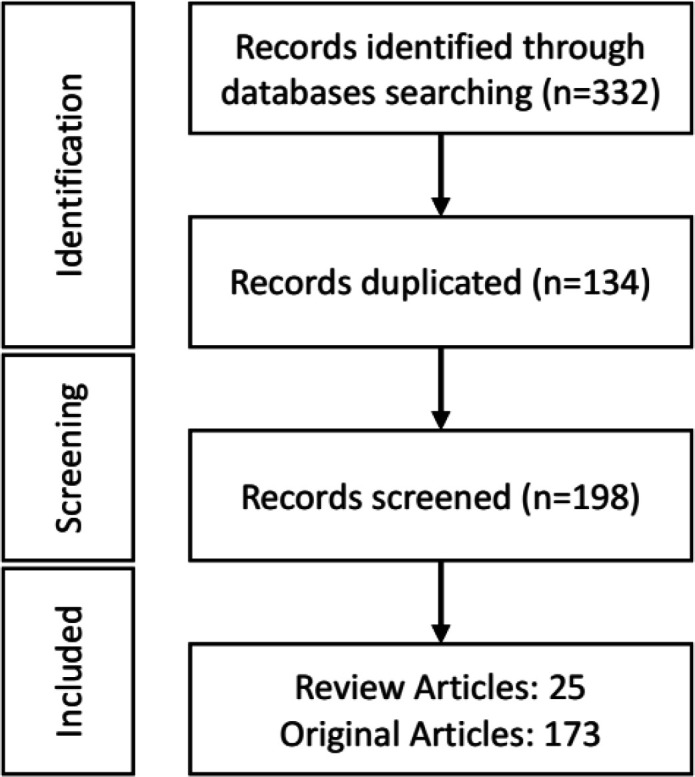
Flowchart of the process for selecting studies for the review

The anti-oxidant effect of *N. sativa* and its derivatives on various disorders were searched using different online databases including; PubMed, Science Direct, Scopus, and Web of Science up to December 31, 2023. Keywords such as *Nigella sativa*, *N. sativa,* black seed or black cumin, thymoquinone or dihydro thymoquinone, anti-oxidants, oxidants, and oxidative stress according to the subject of the article and according to MESH, were used for this review article. Only articles published in the English language and well-known international journals were included in this review article. Totally 327 articles were selected from the above databases, of which 134 articles were duplicates. Therefore, 193 related articles (25 reviews and 168 original) were included in the current review (Figure 2). Eligible articles were selected by two blinded individuals. In cases where the aggregation of opinions about an article was not available, a third party also helped in choosing the article to be included in the study.

## Results

**Lung disorders**

***The effects of various extracts and essential oils of N. sativa***

*In vivo*

The anti-inflammatory and anti-oxidant effects of *N. sativa* oil (4 ml/kg/day for 31 days, orally) in a rat model of allergic asthma were reported. The results showed that in the ovalbumin (OVA)-sensitized, smokeless tobacco (ST)-exposed, and OVA/ST exposed groups, the levels of MDA and carbonyl protein were increased, and the levels of GSH, GSH-Px, CAT, and SOD were decreased in the lung and erythrocytes, which were improved by *N. sativa* treatment (21). On the other hand, the protective effects of macerated hydroethanolic extract of *N. sativa* (100, 200, and 400 mg/kg for 14 days, IP) on lipopolysaccharide (LPS)-induced lung inflammation in rats have also been shown. The results revealed that increased MDA levels in LPS-administered animals, as well as decreased total thiol level, CAT, and SOD activity were improved as a result of *N. sativa* treatment (22). Methanolic extract of *N. sativa* (500 mg/kg/day for 14 days, IP) in bleomycin-induced pulmonary fibrosis in rats also modulated the oxidant/anti-oxidant balance in lung tissue. In the groups treated with *N. sativa* extract, the increased level of lipid peroxidase was reduced and CAT activity was increased (23). The anti-oxidant and anti-lipid peroxidation effects of *N. sativa* oil compared to onion extract have also been reported in nicotine-induced lung damage in rats. The nicotine-treated group showed increased levels of MDA and decreased levels of anti-oxidant markers such as SOD, CAT, lung GSH, and epithelial lining fluid GSH (ELF GSH). Intervention with onion and *N. sativa* oil (0.1 g/kg for 18 weeks, orally) significantly decreased MDA levels and increased SOD, CAT, lung GSH, and ELF-GSH levels, which was predominant in the *N. sativa*-treated group (24). The anti-oxidant effects of *N. sativa* (500 mg/kg, 3 doses, IG) and its essential oil (5 ml/kg, 3 doses, IG) in a model of LPS-induced inflammation in the lung, liver, and kidney tissues were demonstrated in an animal study. Decreased levels of SOD and CAT were observed in the lung tissues of LPS-treated rats. Treatment with *N. sativa* corrected the reduced SOD and CAT activities induced by LPS in the near control group. Increased SOD activity in lung tissue occurred in the *N. sativa* treatment group but this effect was not observed as a result of essential oil treatment. Elevated MDA levels due to LPS induction were also altered as a result of treatment with *N. sativa* and its essential oil (25). In the cecal ligation and puncture (CLP)-induced sepsis model in rats, the anti-oxidant effects of *N. sativa* in preventing lung injury have been shown. Increased levels of LPO and MPO activity as well as decreased GSH levels were observed in the lung tissue of CLP-induced sepsis rats. Treatment with ethanol extract of *N. sativa* (125, 250, and 500 mg/kg, orally) decreased LPO and MPO levels and increased GSH and SOD levels, indicating its anti-oxidative effects (26).

***Effects of TQ***

*In vitro*

The protective effects of TQ on human bronchial epithelial cells exposed to cigarette smoke extract have been demonstrated. The levels of SOD and CAT, and glutathione reductase (GR) activity in CSE-exposed cells were significantly lower than in CSE unexposed bronchial epithelial cells. In BEAS-2B cells under CSE exposure treated with different doses of TQ (20 and 50 µM), it was found that there was a significant increase in anti-oxidant enzyme activities (SOD, CAT, and GR). TQ also significantly increased GSH levels (27). 

The anti-cancer and anti-oxidant effects of TQ have also been reported on A549 non-small cell lung cancer cells exposed to Benzo (a) pyrene. The inflammatory and oxidative stress changes induced by Benzo (a) pyrene, increased levels of oxidative parameters including MDA, GSH, and total oxidant status (TOS)) in A549 cells, which were decreased in TQ-treated cells (5 µM for 48 hr incubation). TQ had no significant effect on total anti-oxidant status (TAS) levels in A549 cells. Interestingly, the anti-oxidant effects of TQ were more pronounced compared to caffeic acid phenethyl ester and resveratrol (28). In another experimental study, TQ (20, 40, and 60 μM) induced oxidative stress by elevating TOS and declining TAC significantly in the A549 lung cancer cell line (29).

*In vivo*

The anti-oxidant effects of TQ (100 mg/kg/day for 14 days, PO) in cyclophosphamide (CPh)-induced lung oxidative damage have also been identified. In the CPh-treated group, a disturbance in TBARS levels and SOD activity, as well as a decreased GSH level, were observed in lung tissue. Treatment with TQ resulted in modulatory effects of oxidant/anti-oxidant parameters (30). Also, in the bleomycin-induced oxidative stress model in rats, it was found that TQ (5 mg/kg/day for 5 weeks, IP) modulates lung injury through its anti-inflammatory and anti-oxidant effects. TQ treatment was found to significantly modify increased lipid peroxides levels and decreased GSH and SOD enzymatic activities in bleomycin-induced lung oxidative stress groups (31). In addition, in bleomycin-induced pulmonary fibrosis in rats, TQ treatment (10 and 20 mg/kg for 28 days, PO) resulted in inflammatory and oxidative changes by increasing GSH and SOD enzyme activity and decreasing MDA levels. The possible mechanism of TQ for modulating oxidative/anti-oxidant activity occurred by increasing the Nrf2 and HO-1 pathway activities as well as reducing the expression of NF-κB (32). The anti-oxidant effect of TQ on PM2.5-induced lung injury in rats was reported. The results identified that TQ treatment (20 and 40 mg/kg for 14 days, IG) reduced oxidative stress parameters such as MDA and increased anti-oxidant activity such as SOD and GSH-PX in rat lung tissue (33). Therapeutic and anti-oxidant effects of TQ have also been identified in Benzo (a) pyrene (B(a)P)-induced lung injury in rats. Oxidative/anti-oxidant imbalance in the (B(a)P)-induced lung injury in rats occurred with increased MDA level but decreased SOD, GSH-Px, CAT, and total anti-oxidant capacity (TAC). Treatment with TQ (50 mg/kg for 8 weeks, orally) significantly reduced MDA levels and increased anti-oxidant marker activities (SOD, GSH-Px, CAT, and TAC) in rat lung tissue (34). The anti-oxidant and anti-inflammatory effects of TQ have also been shown in cecal ligation and puncture (CLP)-induced murine sepsis in various tissues such as the lung, liver, and kidney. The results demonstrated that TQ (1 mg/kg/day for 3 days, IP) had an inhibitory effect on increasing MDA levels and decreasing glutathione levels in liver, kidney, and lung tissues as a result of CLP induction (35). Hyperbaric oxygen (HBO₂)-induced lung injury in rats increased lipid hydroperoxide (LOOH) and SH levels, and TQ treatment decreased oxidative factor levels (36). Paraquat-induced lung fibrosis also reported increased oxidant markers (LPO) and decreased anti-oxidant markers (SOD and CAT) in rat lung tissue, which was reversed by TQ treatment (20 and 40 mg/kg for 28 days, orally), (37). Treatment with TQ significantly modified oxidative/anti-oxidant changes in lung tissue (21).

The above-mentioned experimental studies indicated that *N. sativa* extracts and TQ have protective and therapeutic anti-oxidant effects through reducing oxidants and increasing anti-oxidants in pulmonary disorders induced by various agents such as BLC, CLP, LPS, OVA, nicotine, CPh, PM2.5, Benzo (a) pyrene (B(a)P), or paraquat. The anti-oxidant effects of *N. sativa* and TQ on lung disorders are summarized in Table 1.

**Cardiovascular disorders**

***Effects of various extracts and essential oils of N. sativa***

*In vitro*

A study identified the anti-oxidant effects of water and methanol *N. sativa* extracts on a cellular model of doxorubicin-induced cardio-toxicity (on H9c2 cells). Both water and methanolic *N. sativa* extracts (50 µg/ml) significantly reduced doxorubicin-induced ROS production in H9c2 cells (38).

*In vivo*

The cardiac protective effects of *N. sativa* oil on lead-induced cardiotoxicity in rats have been demonstrated. In lead-treated rats, an increase in MDA levels and a decrease in SOD, GSH, and GSH-Px levels in cardiac tissue were observed. *N. sativa* treatment (4 ml/kg for 1 hr before administration of lead, orally) decreased MDA level and increased SOD, GSH, and GSH-Px levels (39). Elevated MDA levels have also been reported in the myocardial ischemic reperfusion injury model in rats. Treatment with *N. sativa* (800 mg/kg for 12 weeks, orally) significantly reduced MDA levels in the heart tissue of I/R rats. The results of the study indicated that *N. sativa* may have influenced the levels of oxidative factors through an intracellular increase in nicotinamide adenine dinucleotide (NAD+), a factor influencing mitochondrial permeability transition pore (MPTP) (40). The anti-oxidant effect of hydroalcoholic extract of *N. sativa* has also been shown on I/R-induced heart damage in isolated rat hearts. I/R resulted in increased levels of thiobarbituric acid reactive substances (TBARS) and 4-hydroxynonenal (4-HNE) as well as decreased levels of total thiol groups (-SH). Perfused hearts with different concentrations of *N. sativa* extract for 10 min (0.005, 0.02, 0.04, and 0.08 mg/ml) revealed oxidant/anti-oxidant balance correction (decreased 4-HNE and TBARS as well as increased total thiol groups). On the other hand, the reduced activity of SOD and CAT in the I/R-induced heart damage group was improved by treatment with *N. sativa* extract (41). The anti-lipidemic and anti-oxidant effects of methanolic extract (100 mg) and volatile oil (VO, 20 mg) of *N. sativa*, for 30 days, were shown on lipidemic-induced lipid peroxidation in rats. In the hyperlipidemic group, an increase in MDA level was observed, which in all intervention groups was significantly reduced, but was more evident in the methanolic extract group (42). The anti-oxidant and antihypertensive effects of *N. sativa* oil have also been observed in L-NAME-induced hypertensive rats. *N. sativa* oil (2.5 mg/kg/day for 8 weeks, orally) in L-NAME-induced hypertensive rats resulted in a significant cardiac MDA content reduction. Also, increased cardiac NADPH oxidase activity in the L-NAME-treated group was reduced by treatment with *N. sativa* oil. Interestingly, in the *N. sativa*-treated group, increased HO-1 activity in the heart was significantly observed compared to the L-NAME group (43). Improving the effects of *N. sativa* on oxidant/anti-oxidant balance in LPS-induced myocardial fibrosis has also been reported in male rats. Treatment with *N. sativa* hydroalcoholic extract (100, 200, and 400 mg/kg/day, PO) for two weeks improved a significant increase in MDA level and reduction in heart total thiol, SOD, and CAT concentrations in the LPS group (44). Hypertension-induced oxidative stress in rats also showed increased levels of MDA and decreased GSH, and treatment with *N. sativa* oil (0.2 ml/kg for 3 weeks, IP) prevented changes in oxidative stress (45).

*Clinical*

In hypertensive patients (n = 26), administration of *N. sativa* seeds oil for 8 weeks, significantly decreased MDA level with no renal, hepatic, and patient-reported adverse events (46). In patients with coronary artery diseases (n = 60), serum levels of MDA declined significantly following *N. sativa* oil supplementation, while TAC increased (47).

***Effects of TQ***

*In vivo*

TQ has antitoxic effects in the doxorubicin-induced cardiotoxicity in mice, possibly through reduced cell inflammation and oxidative damage. The results showed that doxorubicin-induced cardiotoxicity caused elevated serum levels of LPO and decreased serum levels of GSH, CAT, SOD, GSH-Px, GR, and glutathione-S-transferase (GST). Intervention with TQ (10 and 20 mg/kg for 14 days, PO) decreased LPO and increased anti-oxidant serum markers (GSH, CAT, SOD, GSH-Px, GR, and GST). Therefore, TQ can have therapeutic uses in cardiomyopathy (48). The anti-apoptotic and anti-oxidant effects of TQ on doxorubicin‐cardiotoxicity in rats have also been demonstrated. As a result of doxorubicin-induced cardiotoxicity, elevated TOS levels and decreased TAS levels occurred, and TQ treatment (10 mg/kg/day for 7 days, IP) significantly improved its changes (49). TQ protective effect (9 and 18 mg/kg/day for 21 days, IP) on morphine-induced apoptotic and oxidative damage in the mice heart has been shown. In the morphine-treated group, there was an increase in serum NO level and a decrease in TAC, which was significantly modified in the TQ-treated groups (50). TQ protective effects have also been demonstrated against diazinon cardiotoxicity in rats by oxidant/anti-oxidant balance. Diazinon-induced oxidative damage increased cardiac MDA levels and decreased cardiac GSH levels, as well as decreasing SOD, CAT, GSH-PX, and GST activities. In the TQ-treated group (2.5, 5, and 10 mg/kg/day, for 28 days), a decrease in MDA level and an increase in anti-oxidant marker activities (SOD, CAT, GSH-PX, and GST) were observed in a dose-dependent manner. The overall conclusion of this study was that the cardioprotective TQ effects in the diazinon cardiotoxicity model occurred through cholinesterase activity modification, possibly by its free radical scavenging mechanism (51). In diabetes-caused cardiac myopathy, TQ treatment (50 mg/kg/day for 12 weeks, by gavage,) resulted in decreased plasma and tissue MDA levels (lipid peroxidation index) as well as increased plasma SOD activity and increased cardiac tissue expression. The possible TQ protective effects mechanism was through the up-regulation of Nrf2 in cardiac tissue (52). Interestingly, the prophylactic TQ effects (20 and 50 mg/kg/day for 21 days, orally) on isoproterenol-induced rats myocardial injury have also been suggested through oxidant/anti-oxidant marker modification. Isoproterenol-induced myocardial injury caused elevated heart MDA content as well as decreased heart tissue GSH content. On the other hand, in the myocardial injury group, decreased heart tissue SOD activity was observed. Treatment with TQ, dose-dependently, prevented increased MDA levels and decreased GSH content in the myocardial injury group. Also, the SOD activity improved in the TQ-treated group (53). TQ also improved oxidant/anti-oxidant balance in prilocaine-induced epileptiform activity and cardiotoxicity in rats. Prilocaine treatment decreased TAC and increased ROS/RNS formation in rat heart and brain tissues. TQ treatment (15 mg/kg/day for 3 days, by gavage) decreased ROS/RNS formation and increased TAC in the heart and lung tissue of prilocaine-treated rats (54).

The preventive effects of TQ (2, 5, and 10 mg/kg/day for 3 weeks, IP) on LPS-induced myocardial and perivascular fibrosis damages have also been reported in an animal study. Induction of chronic inflammation with LPS resulted in increased MDA levels and decreased total thiol concentration in the heart tissue of mice. Also in the LPS-treated group, a decrease in SOD and CAT activities was observed. TQ treatment increased SOD and CAT activities in cardiac tissue and decreased MDA levels (55). In addition, the cardioprotective and anti-oxidant effects of TQ in myocardial ischemia/reperfusion (MI/R) injury models of isolated rat hearts were demonstrated. In the MI/R group, elevated levels of hydrogen peroxide (H_2_O_2_) and MDA and decreased SOD activity occurred. TQ treatment (2.5, 5, and 10 μmol/L) significantly improved oxidant/anti-oxidant balance by decreasing MDA and H_2_O_2_ levels and increasing SOD activity in the heart tissue of MI/R rats. The possible mechanism for the cardioprotective effects of TQ was revealed through up-regulation of SIRT1 and inhibition of p53 acetylation (56). 

In the hypercholesterolemic model in rats, it has been reported that TQ (20, 50, and 100 mg/kg for 8 weeks, orally) and TQ-rich fraction (TQRF, 0.5, 1, and 1.5 g/kg for 8 weeks, orally) modulates the oxidant/anti-oxidant status by inhibiting hydroxyl radicals. It was also found that TQRF and TQ treatment resulted in up-regulation of liver tissue SOD1, CAT, and GSH-PX gene expression levels compared to untreated rats. In addition, liver tissue anti-oxidant enzyme levels, SOD1 and GSH-PX, were significantly increased in TQRF- and TQ-treated groups (57, 58). The hypolipidemic and anti-oxidant effects of TQ revealed that TQ treatment (10 mg/kg, twice per day, for 30 days, orally) resulted in inhibition of basal and maximal formation of MDA (59). The anti-oxidant and antilipidemic effects of TQ in the hypercholesterolemic model induced in female rabbits were demonstrated. Increased serum levels as well as aortic tissue of MDA and protein carbonyls were observed in the induced model. TQ treatment (10 and 20 mg/kg/day, through a nasogastric tube for 8 weeks) significantly reduced both serum and aortic tissue concentrations of MDA and protein carbonyls (60). In addition, the TQ protective effects in preventing the development of atherosclerosis in cholesterol-fed rabbits by lowering serum lipid profiles and oxidative stress have been identified. TQ treatment (3.5 mg/day for 4 weeks, orally) corrected elevated TBARS levels as well as decreased GSH levels in cholesterol-enriched chow rabbits (61). Protective effects of TQ have also been reported in cyclosporine and hyperlipidemia-induced atherosclerosis in rabbits. The combined diet of cyclosporine A and hyperlipidemia showed elevated serum and aorta tissue levels of oxidant markers (MDA and protein carbonyl). TQ treatment (10 mg/kg/day for 8 weeks, orally) significantly prevented increase in serum and tissue MDA levels and protein carbonyl in the cyclosporine A and hyperlipidemia-treated group (62). In abdominal aorta ischemia-reperfusion injury in rats, TQ therapeutic effects (20 mg/kg IP, 5 min before reperfusion) on oxidant/anti-oxidant balance have been reported. TQ treatment revealed decreased serum TOS levels as well as oxidative stress index activity. On the other hand, increased serum TAC level was also found as a result of treatment with TQ (63). 

In various cardiovascular disorders induced by LPS, I/R, L-NAME, lead, doxorubicin, morphine, diazinon, isoproterenol, prilocaine, or diabetes, *N. sativa* extracts and TQ modulated the balance between the oxidant/anti-oxidant systems by reducing and increasing oxidant and anti-oxidant markers, respectively. TQ modulated the pro-oxidant/anti-oxidant balance by stimulating the Nrf2 signaling pathway. The anti-oxidant effects of *N. sativa* and TQ, on cardiovascular disorders are summarized in Table 2.

**Gastrointestinal and liver disorders**

***The effects of various extracts and essential oils of N. sativa***

*In vitro*

Protective and anti-oxidant effects of *N. sativa* seed extract on acetaminophen (APAP)-induced hepatotoxicity have been demonstrated. In TIB-73 cells, APAP-induced hepatotoxicity increased ROS production levels. *N. sativa* seed extract treatment (25, 50, 75, and 100 µg/ml) significantly reduced ROS production (64).

*In vivo*

Administration of APAP to rats significantly increased liver homogenate MDA levels compared to the control group. Treatment with *N. sativa* seed extract (100, 300, and 900 mg/kg for 2 weeks, orally) reduced liver tissue MDA levels. In addition, APAP-induced hepatotoxicity in rats decreased anti-oxidant factors, SOD, and GSH, and treatment with *N. sativa* seed extract corrected the changes (64). The anti-oxidant effects of *N. sativa* oil (2 and 4 ml/kg for 28 days, orally) have also been reported in carbon tetrachloride (CCl_4_)-induced hepatotoxicity in rats. Decreased levels of GSH, CAT, MDA, SOD, and NO anti-oxidant enzymes were significantly observed in the CCl_4_-administered group. In a concentration-dependent behavior, the anti-oxidant levels increased significantly in the groups treated with *N. sativa* oil. Also, increased levels of MDA liver tissue due to CCl_4_ administration improved due to 4 ml/kg *N. sativa* oil extract (65). In addition, the protective effects of pretreatment with *N. sativa* oil (2.5 and 5 ml/kg for 3 weeks, orally) on ethanol-induced hepatoxicity in rats have been revealed. Ethanol administration increased hepatic MDA and decreased GSH levels, whereas intervention with 5 ml/kg *N. sativa* oil decreased MDA and increased GSH levels (66). The protective effects of *N. sativa* oil (2 ml/kg for 14 days, orally) on cisplatin (CP)-)-induced gastrointestinal toxicity have also been identified. In the CP-treated intestinal tissue, a significant increase in LPO and a decrease in GSH and total SH were seen. There was also a significant reduction in the activities of SOD, CAT, GSH-Px, GR, GST, and TR in CP-treated rats. However, administration of *N. sativa* oil prevented a decrease in the activities of CAT, GST, GR, SOD, GSH-Px, and TR in CP-treated rats. *N. sativa* oil administration to CP-treated rats also reduced LPO, GSH, and total SH levels (67). The anti-oxidant effects of *N. sativa* oil have also been shown in cyclophosphamide-induced hepatotoxicity. Decreased GSH, SOD, and CAT levels in cyclophosphamide-treated rats were prevented by administration of *N. sativa* oil (50 and 200 mg/kg for 15 days, orally) (68). The protective effects of *N. sativa* seeds on lead acetate-induced oxidative liver injury in rabbits have also been identified. Lead-treated rabbits showed increased levels of MDA and decreased GSH as well as GSH-Px and GST activities. The use of *N. sativa* seed supplement (20 g/kg diet for 8 weeks, orally) reduced MDA levels and improved GSH, GSH-Px, and GST contents (69). In addition, increased MDA and GSH levels, as well as decreased GSH-Px, CAT, and SOD enzyme activities in aluminum chloride-induced oxidative injury in the liver have been observed in rats. *N. sativa* oil pretreatment (2 ml/kg for 8 weeks, by gavage) in the aluminum chloride-treated rats, prevented the oxidant/anti-oxidant imbalance in liver tissue (70). Potassium bromate-induced oxidative stress in rats has been shown to have a highly pronounced oxidant/anti-oxidant imbalance. In potassium bromate-treated rats, MDA levels increased and TAC, GST, GR, GSH-Px, SOD, α-Tocopherol, and γ-Tocopherol levels and activity decreased. Intervention with both *N. sativa* fixed (4%) and essential oil (3%) diets over a period of 56 days prevented the aforementioned changes (71). The hepatoprotective effects of *N. sativa* oil against oxidative stress in thioacetamide-induced liver cirrhosis have also been demonstrated in rats. Thioacetamide-treated rats showed an increase in TBARS and a decrease in CAT, SOD, GSH-Px, and GSH; and *N. sativa* oil treatment (5 and 10 ml/kg for 8 weeks) prevented changes, especially in high concentrations (72).

The anti-oxidant effects of *N. sativa* oil on ischemia/reperfusion (I/R)-induced gastric lesions have also been demonstrated. It has been shown that the production of free radicals increases in I/R. In I/R-induced gastric lesion rats, elevated levels of lipid peroxide (LPX) occurred while GSH and SOD decreased. Treatment with *N. sativa* oil (2.5 and 5 ml/kg, 30 min before I/R, orally) and TQ (5, 20, 50, and 100 mg/kg, 30 min before I/R, orally) prevented a decrease in GSH and SOD while modulating elevated LPX levels (73). In addition, in intestinal I/R injury in rats, the preventive effects of *N. sativa* have been shown. Increased levels of TOS, oxidative stress index (OSI), and MPO were evident in ileum-induced I/R rats, while levels of TAC and CAT decreased. Pretreatment with *N. sativa *volatile oil (0.2 ml/kg, IP) prevented all of the above changes including decreasing TAC and CAT as well as increasing TOS, OSI, and MPO (74). *N. sativa* modulatory effects on oxidant/anti-oxidant markers in rat hepatic ischemia-reperfusion injury have also been reported. Pretreatment with *N. sativa* (0.2 ml/kg, IP) before I/R induction in rat hepatic tissue inhibited the increase of oxidants (TOS, MPO, and OSI) and decreased anti-oxidants markers (CAT and TAC) (75). 

In a rat model of acrylamide-induced liver toxicity, administration of *N. sativa* essential oil (10 mg/kg, IP for 15 days) significantly reduced oxidative stress and pro-inflammatory cytokine levels in liver tissues (76). In a rat model of arsenic-induced redox imbalance, *N. sativa* oil treatment improved the anti-oxidant status and energy metabolism, indicating the ability of the intestine to protect against free radical-mediated arsenic toxicity in the intestine (77). The combination of 2% nigella with 7% frying oils in rats’ diets revealed an intense reduction in oxidative stress and ameliorated the levels of the majority of hepatic biomarkers (78).

*Clinical*

*N. sativa* supplementation in patients with ulcerative colitis (n = 46) reduced MDA levels compared to placebo. There was no significant difference between the two groups in serum total anti-oxidant capacity (79).

***Effects of TQ***

*In vivo*

The anti-oxidant effects of TQ have been extensively studied in the gastrointestinal tract. The protective effects of TQ on acrylamide-induced oxidative damage in liver tissue have been revealed. Cadmium-induced liver oxidative stress in mice under *in vitro* conditions showed increased levels of carbonyl protein and decreased GSH, while TQ treatment (10 µM, IP) increased anti-oxidant activity (80).

After acrylamide-induced toxicity in rat liver tissue, increased levels of NO and MDA were seen, while GSH concentration and SOD, GSH-Px, and CAT activities decreased. Pretreatment with TQ (10 and 20 mg/kg for 21 days, orally) in acrylamide-intoxicated rats decreased NO and MDA as well as increasing GSH concentration and anti-oxidant enzyme activity (81). The protective effect of TQ against arsenic-induced hepatotoxicity in female rats has also been revealed. As a result of arsenic intoxication, hepatic oxidative damage was revealed with a significant decrease in anti-oxidant parameters (CAT, SOD, GR, GSH-Px, and GSH) that were accompanied by a concomitant increase in MDA levels. The disturbances of the observed anti-oxidant enzymes were prevented as a result of TQ pretreatment (10 mg/kg for 28 days, orally) (82). The anti-oxidant effects of TQ and curcumin have also been shown in gentamicin-induced liver injury. As a result of gentamicin administration, the MDA level increased significantly while GSH level and GSH-Px and SOD activities decreased. Curcumin and TQ treatment (20 mg/kg for 21 days, orally) significantly reduced MDA and increased GSH levels and SOD and GSH-Px activities in the gentamicin-administered animals, with curcumin effects more pronounced (83). TQ modulator’s effects on oxidant/anti-oxidant imbalance in bisphenol A-induced hepatotoxicity have also been observed in male rats. In bisphenol A-treated rats, there was an increase in liver tissue MDA and LPO as well as a decrease in TAC and GSH levels along with a decrease in the anti-oxidant enzyme activities (GSH-PX, SOD, GST, and CAT). Intervention with TQ (10 mg/kg for 5 weeks, orally) prevented the above oxidant/anti-oxidant imbalances, as MDA and LPO levels decreased and anti-oxidant levels and activity increased (84). In addition, TQ post-treatment led to anti-oxidant effects in atorvastatin-induced hepatic injury in rats. Serum and hepatic homogenized tissue of atorvastatin-treated rats showed increased levels of MDA and protein carbonyl as well as decreased levels of GSH and CAT. After 8 weeks of atorvastatin-induced liver injury, TQ treatment (30 mg/kg for 10 consecutive days, orally) resulted in a detectable decrease in MDA and protein carbonyl as well as increased GSH and CAT activity (85). Protective effects of TQ have also been reported in 2,3,7,8-tetrachlorodibenzo-p-dioxin (TCDD)-induced hepatotoxicity in rats. TCDD administration in rats increased MDA and TOS as well as decreased GSH, CAT, SOD, and TAC levels in liver tissue. TQ treatment (50 mg/kg for 30 days, orally) significantly prevented changes in rat liver tissue by TCDD administration and balanced oxidant/anti-oxidant factors (86). In addition, the protective effects of TQ on CCl_4_-induced liver injury in mice have been identified. A significant decrease in anti-oxidant enzyme activity (SOD, GSH-Px, and CAT), as well as a decrease in TAC, was seen in CCl_4_-treated mice. TQ treatment (10 mg/kg for 12 weeks, orally) in CCl_4_-treated mice significantly prevented the above anti-oxidant changes (87). TQ and its nanoparticle form (NTQ particles), showed a hepatoprotective effect against diazinon-induced hepatic injury. The mechanisms underlying this effect may involve counteracting diazinon-induced oxidative injury markers, such as significant increase in NO, ROS, H_2_O_2_, MDA, and protein carbonyl, as well as increased apoptotic markers (88).

The protective role of TQ in paraquat-induced hepatoxicity has also been observed in mice. In paraquat-treated mice, increased LPO levels and decreased TAC were observed in liver tissue, and TQ pre-treatment (5, 10, and 20 mg/kg for 4 days, orally) normalized the above changes. In addition, there was a decrease in the SOD and CAT enzyme activities. TQ treatment with a concentration of 10 mg/kg resulted in a modification of SOD activity (89).

In the nonalcoholic fatty liver disease model in rats, the TQ protective effects through oxidant/anti-oxidant balance have also been observed. In the nonalcoholic fatty liver disease model, elevated MDA and decreased TAC levels occurred. TQ intervention caused dose-dependent increased TAC levels and decreased MDA levels (10 and 20 mg/kg for 6 weeks by gavage) (90). TQ has also been shown to have anti-oxidant effects on LPS-induced liver fibrosis in rats. In LPS-treated rats, liver fibrosis and oxidant/anti-oxidant imbalance (elevated MDA and decreased CAT and SOD) were observed. TQ treatment (2, 5, and 10 mg/kg for 3 weeks, IP) modified inflammatory-induced liver fibrosis by affecting oxidant/anti-oxidant balance (reduced MDA and increased activity of SOD and CAT enzymes) (91). 

The protective effects of pretreatment with TQ on partial hepatectomy (PH) under I/R in rats have also been observed. As a result of PH under I/R, the liver injury occurred through various pathways such as increasing oxidants (MDA and conjugated dienes) and decreasing anti-oxidants (GSH-Px, SOD, CAT, and sulfhydryl proteins). Pretreatment with TQ (30 mg/kg for 10 days, orally) prevented mentioned changes (92). 

Intestinal I/R injury is a condition that results in a disturbance of the oxidant/anti-oxidant balance in the gastrointestinal tract. In the animal intestinal I/R model, increased SOD and GSH-Px activities but increased MDA levels were observed. Pretreatment with TQ (50 mg/kg 30 min before the ischemia period, IP) improved all three markers (SOD, GSH, and MDA) (93). The anti-oxidant effects of TQ on partial hepatic warm I/R in rats have been revealed. In the I/R group, increased levels of liver tissue MDA and CD concentrations, as well as decreased levels of sulfhydryl proteins, were observed. Pretreatment with TQ (30 mg/kg for 10 days, orally) prevented oxidative and anti-oxidant changes in I/R-treated rat liver tissue (94). Oxidative and anti-oxidant changes have also been revealed in animal studies on I/R-induced gastric mucosal injury in rats. In I/R-induced gastric mucosal injury in rats increased levels of MDA and MPO as well as decreased SOD, GSH, and total NO occurred. TQ treatment (10 and 30 mg/kg, 30 min before I/R induction) prevented increase in MDA and MPO as well as decrease in SOD, GSH, and total NO levels in I/R-treated rats (95). In another study, the TQ protective effects (50 mg/kg, orally, 30 min, 24 hr, and 48 hr prior to the surgical procedure) on I/R-induced intestinal injury in rats through oxidant/anti-oxidant balance were reported. In I/R ileum tissue, an increase in MDA and MPO levels and a decrease in GSH activity were seen, and TQ pretreatment prevented these changes (96). The hepatoprotective effects of TQ through oxidant/anti-oxidant modulation in hepatic I/R injury in rats have also been investigated. Increased MDA levels and decreased GSH in I/R treated rats were inhibited by TQ aqueous solution pretreatment (5, 20, and 50 mg/kg for 10 days, orally) (97). In addition, another study found that pretreatment with TQ (20 mg/kg for 10 days, orally) prevented increased hepatic lipid peroxidation (MDA) and decreased GSH in I/R rats (98). 

The protective effects of TQ on chronic cyclosporine A (CsA)-induced hepatotoxicity as well as I/R-induced acute liver injury in rats were reported. In CsA treatment and liver I/R groups, an increase in MDA level and a decrease in GSH level and SOD activity were observed, where TQ treatment (10 mg/kg for 28 days and 1 day by gavage for CsA and I/R treated groups, respectively) resulted in reversal of oxidative stress markers (99). Increased MDA levels and decreased GSH, GST, CAT, SOD, and GSH-Px levels have been shown in cisplatin-induced hepatotoxicity in rats. TQ treatment (500 mg/kg for 30 days, orally) resulted in a detectable decrease in MDA and an increase in anti-oxidant levels (100). Reduced levels and activities of anti-oxidants have been shown in the lead-induced liver oxidative damage in rats. TQ treatment (5 mg/kg for 5 weeks, orally) increased anti-oxidant levels and activities (SOD, GSH-Px, CAT, GR, and GSH) in lead-treated rats (101). In acetaminophen-induced hepatotoxicity in rats, serum and liver tissue levels of GSSG, SOD, and MDA increased while GSH-Px decreased. TQ treatment (5 mg/kg for 24 hr, orally) corrected oxidant/anti-oxidant changes (102). Elevated levels of hydroxyproline (HP) and MDA as well as decreased SOD and GSH-Px occurred in cholestatic rats with liver injury. TQ treatment (25 and 50 mg/kg for 15 days, by gavage) significantly improved oxidative (MDA and HP) and anti-oxidant (SOD and GSH-Px) markers (103). In methotrexate-induced liver toxicity in rats, elevated NO and MDA levels and decreased CAT and GSH activities were corrected by TQ treatment (10 mg/kg for 10 days, orally) (104). In tamoxifen-induced liver injury in female rats, increased LPO levels and decreased SOD and GSH levels have been reported. TQ pretreatment (50 mg/kg for 20 days, orally) significantly modified oxidant/anti-oxidant changes (105). Aflatoxin B1-induced liver toxicity in mice revealed an increase in MDA and a decrease in GSH levels. Pretreatment with TQ (4.5, 9, and 18 mg/kg for 3 days, orally) significantly reduced MDA and increased GSH levels (106). Decreased enzymatic activity of SOD and CAT has been shown in hyperbilirubinemia and cyclophosphamide-induced hepatotoxicity in mice. Treatment with TQ or liposomal formulation of TQ (5 and 10 mg/kg for 3 days, IP) increased the activities of the anti-oxidant enzymes SOD and CAT (107). In anti-tuberculosis drug-induced liver damage in rats, increased LPO levels and decreased SOD and CAT levels were found. TQ treatment (10, 20, and 40 mg/kg for 8 weeks, orally) corrected oxidative stress changes in a dose-dependent behavior (108). Acetaminophen-induced oxidative stress in mice liver increased total nitrate/nitrite and LPO levels and decreased GSH levels. Treatment with TQ (0.5, 1, and 2 mg/kg for 5 days, orally) reversed the changes in a dose-dependent manner (109). Titanium dioxide nanoparticle-induced toxicity in rat liver increased LPO as well as decreasing GSH and TBARS in liver tissue. TQ treatment (20 mg/kg for 6 weeks, orally) resulted in a significant decrease in LPO but an increase in GSH and TBARS, which had significant effects compared to avenanthramides (110). Altogether, head irradiation-induced oxidative stress in rats, increased total oxidant status levels, lipid hydroperoxide level, and oxidative stress index, as well as decreased total anti-oxidant status levels, sulfhydryl levels, and paraoxonase (PON) activity were reported. TQ treatment (50 mg/kg for 30 days, IP) significantly improved oxidant/anti-oxidant balance in the irradiated (IR) group (111). In I/R-induced liver injury in rats, increased MDA, MPO, and NO levels as well as decreased GSH levels in liver tissue have been reported. Pretreatment with TQ (20 mg/kg for 10 days, orally) resulted in increased GSH and decreased MDA, NO, and MPO (98). 

From the above studies, it is shown that *N. sativa* extracts and TQ lead to protective and therapeutic effects via modulating the oxidative/anti-oxidant pathways in various gastrointestinal and liver disorders induced by APAP, lead, potassium bromate, I/R, CCl_4_, ethanol, CP, CsA, cyclophosphamide, aluminum chloride, titanium dioxide, acrylamide, arsenic, gentamicin, bisphenol A, atorvastatin, TCDD, paraquat, LPS, cadmium, cholestatic, methotrexate, tamoxifen, aflatoxin B1, or hyperbilirubinemia. The anti-oxidant effects of *N. sativa* and TQ on gastrointestinal and liver disorders are summarized in Table 3.

**Renal disorders**

***The effects of various extracts and essential oils of N. sativa***

*In vivo*

The effects of *N. sativa* hydroalcoholic extract in preventing kidney oxidative damage in diabetic rats have been demonstrated. Elevated levels of MDA and decreased levels and activities of anti-oxidants such as total thiol groups, SOD, and CAT were observed in the kidneys of diabetic rats. *N. sativa* treatment (200 and 400 mg/kg for 6 weeks, orally) in diabetic rats resulted in improved anti-oxidant status and decreased MDA in renal tissue (112). The anti-oxidant and protective effects of *N. sativa* on cisplatin-induced nephrotoxicity have been demonstrated in an animal study. In cisplatin-treated rats, increased levels of MDA and decreased CAT, GSH-Px, and SOD were seen. Comparative intervention with powder (3 g/kg/day), oil (2 g/kg/day), and extract (0.5 g/kg/day for 60 days, by gavage) of *N. sativa* in cisplatin-treated rats caused an oxidative stress change that was significant in the extract and oil groups. Interestingly, the effects of *N. sativa* extract were more noticeable than *N. sativa* oil on oxidant/anti-oxidant balance (113).

*N. sativa* has been shown to prevent renal damage in the renal I/R injury model by preventing oxidant/anti-oxidant imbalance. In I/R-treated rats, increased MDA levels and decreased anti-oxidant enzyme activities (SOD, CAT, GSH, and GSH-Px) were observed in renal tissue. Pretreatment with macerated extract of* N. sativa* (0.5, 1, and 2% in the diet, for 3 weeks), concentration-dependently, prevented the increase of MDA and improved the anti-oxidant enzyme activities (114). In addition, renal-protective effects of *N. sativa* have been shown to establish an oxidant/anti-oxidant balance in renal I/R injury rats. Increase in TOS, MPO, and OSI but decreased TAC values and CAT activity were observed in the renal tissue of I/R-treated rats. Pretreatment before induction of I/R in animals with volatile oil *of N. sativa* (0.2 ml/kg, IP) prevented an increase in oxidants and a decrease in anti-oxidant markers (115). Another study of I/R injury in rat kidneys reported increased levels of MDA, protein carbonyl, and NO, as well as decreased SOD and GSH-Px. Pretreatment (0.3 ml/kg for 7 days, by gavage) and after treatment (0.6 ml/kg, by gavage) with *N. sativa* prevented oxidative and anti-oxidant changes. The *N. sativa* treatment effects after kidney injury induction were more detectable than treatment seven days before kidney injury induction with I/R (116). Pretreatment (150 and 300 mg/kg, i.v.) and post-treatment (150 and 300 mg/kg, i.v.) with *N. sativa* hydroalcoholic extract after I/R induction showed that both methods (pre-and post-treatment) reduced MDA and renal thiol content modification (117). 

The anti-oxidant effects of *N. sativa* hydroalcoholic extract on renal oxidative damage associated with propylthiouracil (PTU)-induced hypothyroidism have been reported during neonatal and juvenile growth. In PTU-treated animals, increased levels of renal MDA and total thiol concentration, as well as decreased SOD and CAT activities were seen. *N. sativa* administration (100, 200, and 400 mg/kg for 8 weeks, orally), decreased MDA levels and increased thiol, SOD, and CAT activities in renal tissue, which was more detectable at its high concentrations (118).

CP-induced oxidative renal injury in rats has been shown to increase MDA and decrease GSH and total SH. Administration of *N. sativa* oil (2 ml/kg for 14 days) in CP-treated animals, prevented an increase in MDA and a decrease in GSH and total SH (119). The anti-oxidant effects of oral *N. sativa* oil on radiation-induced oxidative stress in rat renal tissue have also been revealed. The results showed that oxidative parameters such as lipid hydroperoxide, total oxidant status, and OSI in the irradiation-treated rats group significantly increased compared to the control group. Intervention with *N. sativa* oil (1 g/kg for 10 days, gavage) significantly prevented the increase of oxidative parameters. On the other hand, in relation to the measured anti-oxidant parameters such as arylesterase, paraoxonase, sulfhydryl group, total anti-oxidant status, and ceruloplasmin in renal tissue, it was found that the parameters of paraoxonase, total anti-oxidant status, and ceruloplasmin in radiation-treated rats were significantly reduced compared to the control group. *N. sativa* oil treatment was only able to prevent paraoxonase and ceruloplasmin depletion under radiation-induced oxidative stress (120). 

***Effects of TQ***

*In vivo*

Increased levels of MDA and decreased levels of GSH and Total-SH were indicated in CP-induced renal oxidative injury. TQ pretreatment (0.5, 1.5, and 3 mg/kg for 14 days, orally), decreased MDA level and increased GSH and Total-SH activities, where the effects of TQ on the renal cortex were more pronounced than those of the medulla (119). The renal-protective effects of TQ against gentamicin (GM)-induced renal oxidative stress have also been demonstrated in rats. In GM-induced nephrotoxicity animals, TQ treatment (20 mg/kg every other day for 21 days, orally) prevented an increase in MDA and a decrease in GSH content as well as decreased anti-oxidant enzyme activates (SOD and GSH-Px) (121). Renal tissue oxidant/anti-oxidant imbalance has also been reported in cadmium-induced nephrotoxicity in animal studies. In cadmium-treated rats, there was an increase in MDA levels and a decrease in CAT, SOD, and GSH-Px activities, but TQ treatment (50 mg/kg for 30 days, orally) corrected these changes (122). The protective effect of TQ on sodium nitrite-induced renal toxicity in rats was indicated. In sodium nitrite-treated rats, renal MDA increased but GSH decreased, and TQ treatment (25 and 50 mg/kg for 3 months, orally) in dose-dependent manner decreased MDA and increased GSH levels (123). In sodium arsenite-induced nephrotoxicity in female rats, increased MDA and NO levels as well as decreased GSH levels and the activities of anti-oxidant enzymes (SOD, CAT. GSH-Px, and GR) have been shown. Post-treatment with TQ (10 mg/kg for 28 days, orally) significantly reduced MDA and NO but increased GSH levels and all anti-oxidant enzymes (increased SOD, CAT. GSH-Px, and GR activity) (124). Acrylamide-induced nephrotoxicity in rats showed an increase in MDA and NO levels and a decrease in GSH, GSH, Px, SOD, and CAT activities. TQ intervention (10 and 20 mg/kg for 21 days, orally) resulted in oxidant/anti-oxidant balance improvement in acrylamide-induced nephrotoxic rats (81). Oxonic acid (OA)-induced hyperuricemia in rats is also a model used to study oxidant/anti-oxidant balance. It has been shown that in the OA-induced hyperuricemia rats model, there was a decrease in the activities of key anti-oxidant enzymes such as CAT, SOD, GR, GSH-Px, and aconitase. TQ treatment with concentrations of 10 and 20 mg/kg (12 weeks, orally) increased anti-oxidant activities, which was very evident in concentrations of 20 mg/kg (125). The diclofenac-induced acute kidney injury in rats also showed increased levels of MDA and decreased GSH, TAC, and CAT. TQ (20 mg/kg for 21 days, orally) treatment decreased MDA and increased anti-oxidant markers in renal tissue (126). In addition, gentamicin-induced acute renal failure in rats also increased MDA and decreased GSH, SOD, and GSH-Px in renal tissue. Post-treatment with different concentrations of TQ (10, 20, and 30 mg/kg for 28 days, orally) corrected oxidant/anti-oxidant markers that were more pronounced at high concentrations (127). In a study, arsenic-induced nephrotoxicity in rats increased oxidative factors such as MDA and decreased anti-oxidant markers such as SOD, CAT, and GSH-Px in renal tissue. Intervention with TQ (10 mg/kg for 15 days, IG) decreased MDA levels and increased SOD, CAT, and GSH-PX activities (128). In 2, 3, 7, 8-tetrachlorodibenzo-p-dioxin induced nephrotoxicity in rats, oxidant/anti-oxidant imbalance in renal tissue was also detected, as MDA and TOS levels were increased and anti-oxidant markers (GSH, CAT, SOD, and TAS) were reduced. TQ treatment (50 mg/kg for 30 days, orally) in the nephrotoxicity-treated group prevented the increase of oxidative parameters and decreased anti-oxidant markers (129). TQ protective effects against renal fibrosis in rats through oxidant/anti-oxidant balance have also been demonstrated. In the LPS-induced renal fibrosis, increased MDA levels and decreased total thiol groups as well as decreased SOD and CAT activities were identified. TQ treatment (2, 5, and 10 mg/kg for 21 days, IP) prevented oxidant/anti-oxidant changes in the LPS-treated group (130). Elevated MDA levels and decreased SOD and GSH-PX activity have been identified as markers of vancomycin-induced nephrotoxicity in rats. Treatment with TQ (10 mg/kg for 8 days, IP) has been shown to prevent oxidative enhancement (MDA) and decreased anti-oxidant activity (SOD and GSH-PX) (131). The renal-protective effects of TQ on contrast-induced nephropathy in rats were indicated by establishing oxidant/anti-oxidant balance in renal tissue. TQ treatment (1 and 1.75 mg/kg for 4 days, IP) in the contrast-treated group caused decreased serum MDA levels and increased serum SOD levels (132). CP-induced oxidative renal injury in rats has been shown to increase MDA and decrease GSH and total SH. Administration of TQ (1.5 mg/kg for 14 days) in CP-treated animals decreased MDA levels but increased GSH and total SH levels (119). In a rat model of carfilzomib-induced renal impairment, TQ administration significantly mitigated the negative effects of carfilzomib treatment on the anti-oxidant defense system (133).

The anti-oxidant effects of TQ on radiation-induced oxidative stress in rat renal tissue have also been revealed. The results showed that oxidative parameters such as lipid hydroperoxide, total oxidant status, and OSI in the irradiation-treated rats group increased significantly compared to the control group. Intervention with TQ (50 mg/kg for 10 days, IP) significantly prevented the increase of oxidative parameters. On the other hand, in relation to the measured anti-oxidant parameters such as arylesterase, paraoxonase, sulfhydryl group, total anti-oxidant status, and ceruloplasmin in renal tissue, the parameters of paraoxonase, total anti-oxidant status, and ceruloplasmin in radiation-treated rats were significantly reduced compared to the control group. TQ treatment was only able to prevent paraoxonase and ceruloplasmin depletion under radiation-induced oxidative stress (120).

In the I/R-induced renal injury in rats, increased anti-oxidant marker (MDA) and decreased anti-oxidant marker (GST) were reported, but TQ pre-treatment (10 mg/kg for 10 days, orally) reversed increased MDA level and decreased GST activity (134). The protective effects of TQ on chronic CsA-induced nephrotoxicity and I/R-induced acute renal injury in rats were reported. In CsA treatment and renal I/R groups, elevated MDA and decreased GSH levels and SOD activity were observed, and TQ treatment (10 mg/kg for 28 days and 1 day by gavage for CsA and I/R treated groups, respectively) resulted in reversal of oxidative stress markers (99). The protective effects of TQ on renal tissue have also been revealed in the propylthiouracil-induced hypothyroid rat model by establishing oxidant/anti-oxidant balance markers. In the propylthiouracil-treated group, TQ treatment (50 mg/kg for 4 weeks, by gavage), decreased MDA levels and increased GSH-Px, SOD, and CAT enzymatic activities (135).

The above-mentioned experimental studies indicated that *N. sativa* extracts and TQ have protective and therapeutic effects on kidney oxidative stress by reducing oxidants and increasing anti-oxidants in various renal disorders induced by I/R, cisplatin, PTU, gentamicin, vancomycin, diclofenac, cyclosporine A, propylthiouracil, acrylamide, sodium nitrite, sodium arsenite, oxonic acid, cadmium, arsenic, tetrachlorodibenzo-p-dioxin, LPS, contrast, or radiation. The anti-oxidant effects of *N. sativa* and TQ on renal disorders are summarized in Table 4.

Central and peripheral nervous system disorders

***The effects of various extracts and essential oils of N. sativa***

*In vivo*

In the pentylenetetrazol-treated mice, increased MDA, xanthine oxidase (XO), and adenosine deaminase (ADA) levels, as well as decreased SOD and GSH-Px activity, were observed in brain tissue, and *N. sativa* oil pretreatment (12 ml/kg for 21 days, orally) prevented the above changes (136). In CCl_4_-treated rats, elevated MDA and NO levels occurred, but *N. sativa* oil pretreatment (2 and 4 ml/kg for 28 days, orally) in high concentrations prevented these changes. CCl_4_-treated animals also experienced a decrease in CAT and SOD activities and GSH levels, and *N. sativa* oil inhibited the mentioned changes in brain tissue (65). The anti-oxidant and antinociceptive effects of *N. sativa* oil have been revealed in two models of acute (carrageenan-induced) and sub-acute inflammation (complete Freund’s adjuvant-induced). In Freund’s adjuvant (FA)-treated female rats, oxidant/anti-oxidant imbalance was found as serum levels of MDA and oxidized glutathione (GSSG) increased and anti-oxidant markers GSH, SOD, and hydrogen donor capacity (DH) decreased. In the FA-treated rats, pre-and post-treatment with *N. sativa* oil (4 ml/kg for 7 days, orally) corrected MDA, GSSG, GSH, DH, and SOD alternation to establish oxidant/anti-oxidant balance (137). Anti-oxidant effects of *N. sativa* aqueous and hydroalcoholic extracts (400 mg/kg for 7 days, orally) in a model of cerebral ischemia induced by middle cerebral artery occlusion (MCAO) have also been shown. In the MCAO-treated rats, increased levels of TBARS and decreased GSH as well as decreased activity of anti-oxidant enzymes SOD and CAT were observed. Pretreatment with *N. sativa* extracts decreased TBARS and increased glutathione, SOD, and CAT levels (138). Neuroprotective effects of *N. sativa* (chloroform and petroleum ether extracts) on stroke models in rats through oxidant/anti-oxidant balance markers have also been reported. In the MCAO-treated rats, both extracts of *N. sativa* pretreatment (400 mg/kg for 7 days, orally) caused decreased TBARS and increased GSH levels as well as decreased SOD and CAT activities (139). In experimental head trauma in rats, the anti-oxidant effects of *N. sativa* have been identified by modulating the oxidant/anti-oxidant balance. Under head trauma conditions increased MDA and decreased TAS levels indicated that *N. sativa* treatment corrected these changes (140). Cerebral hypoperfusion induced by permanent occlusion of bilateral common carotid arteries (POBCA) in rats has been shown to elevate MDA levels and decrease SOD enzymatic activity in the hippocampal portion. Treatment with *N. sativa* hydroalcoholic extract (100, 200, and 400 mg/kg for 10 days, IP), decreased MDA levels and increased SOD activity (141).

***Effects of TQ***

*In vitro*

The model of brain ischemia and neurodegenerative disorders has been used in *in vitro* studies via serum/glucose deprivation (SGD)-induced cell death in cultured PC12 cells. Elevated ROS levels were observed in SGD-treated cells, which were prevented by pretreatment with TQ (1.17-150 µM for 2 hr) and *N. sativa oil* (15.62-250 µg//ml for 2 hr) (142). Inhibitory effects of TQ on LPS-induced neuroinflammation have been reported in the BV2 mouse microglial cell line by activating anti-oxidant mechanisms. TQ (2.5–10 µM for 12 hr) has been shown to activate anti-oxidant pathways in the BV2 microglial by increasing the expression of Nrf2, HO-1, ARE, and NQO1 (143). In addition, in sodium nitrite-induced oxidative brain damage, increased MDA level and decreased GSH activity were observed, which TQ treatment (*in vitro*: 0, 10, 50, and 100 µM) led to the correction of the mentioned changes in a dose-dependent manner. On the other hand, in sodium nitrite-treated brain cells, there was a decrease in Nrf2 expression level and cytochrome c oxidase activity, and TQ treatment reversed all alternation in a concentration-dependent manner (144). Elevated levels of MDA, superoxide, NO, hydrogen peroxide, and lipid hydroperoxide, as well as decreased GSH, SOD, and CAT activities, were seen in LPS/IFNγ or H_2_O_2_-activated BV-2 microglia, which changes were improved by treatment with TQ (0-40 µM) (145).

*In vivo*

The neuroprotective effects of *N. sativa* oil and TQ on radiation-induced oxidative stress in brain tissue have also been revealed. In irradiation-treated rats, decreased total superoxide scavenger activity (TSSA), GST and GSH-Px activities, non-enzymatic superoxide scavenger activity (NSSA), SOD, and paraoxonase (PON) activities, as well as decreased TAS and total sulfhydryl (-SH) group were observed in brain tissue. On the other hand, in irradiation-treated animals, XO activity, TOS, OSI, and LOOH levels showed a significant increase. Treatment with *N. sativa* (1 g/kg for 10 days, orally) and TQ (50 mg/kg for 10 days, IP) corrected all oxidant/anti-oxidant changes except GST and GSH-Px activities (146). Cerebral hypoperfusion induced by POBCA in rats has been shown to elevate MDA levels and decrease SOD enzymatic activity in the hippocampal portion. Treatment with TQ (10, 20, and 40 mg/kg for 10 days, IP) decreased MDA level and increased SOD activity (141). 

Increased MDA and NO levels and decreased GR, SOD, GSH, CAT, and GSH-Px activates were seen in arsenate-treated rats in the cerebral cortex, cerebellum, and brain stem. Post-treatment with TQ (10 mg/kg for 21 days, IP) suppressed arsenic-induced neurotoxic effects by reducing oxidants (MDA and NO) and increasing anti-oxidant markers (GSH, GR, GSH-Px, SOD, and CAT) (147). The neuroprotective effects of TQ in the Parkinson’s disease (PD) animal model have also been demonstrated through oxidant/anti-oxidant balance. In PD-treated rats, an increase in prooxidant-anti-oxidant balance (PAB) was observed, and treatment with TQ (7.5 and 15 mg/kg/day, PO) significantly reduced PAB (148). 

In acrylamide-treated rats, increased levels of MDA and NO but decreased GSH, GSH-Px, SOD, and CAT were observed in brain tissue. TQ treatment (10 and 20 mg/kg for 21 days, PO) ameliorated all acrylamide-induced changes, which were seen at high concentrations of TQ (81). The TQ-improving effects (2.5, 5, and 10 mg/kg for 8 weeks, IP) on D-galactose (D-gal)-induced rat memory impairments have been demonstrated through various pathways such as oxidative stress. Elevated hippocampal tissue MDA and decreased GSH levels occurred in D-gal-treated rats, treatment with 2.5 mg TQ showed inhibitory effects (149). In cisplatin-induced neurotoxicity in rats, elevated levels of MPO and 8-isoprostane, as well as decreased SOD activity, were found, and TQ treatment (20 mg/kg three times a week, PO) corrected the above changes (150). Increased levels of MDA, but decreased GSH-Px, SOD, and CAT activities have been reported in lead-induced brain oxidative damage in rats. Treatment with TQ (10 mg/kg for 20 days, orally) decreased MDA levels and increased SOD, CAT, and GSH-Px activities in brain tissue (151). In addition, in sodium nitrite-induced oxidative brain damage, increased MDA levels and decreased GSH activity were observed, and TQ treatment (*in vivo*: 50 mg/kg for 12 weeks, orally) led to correction of these changes in a dose-dependent manner. In sodium nitrite-treated animals, there was a decrease in Nrf2 expression levels and cytochrome c oxidase activity, and TQ treatment reversed all alternation in a concentration-dependent manner (144). In the subarachnoid hemorrhage induced by cerebral vasospasm in rats, increased TOS and OSI levels and decreased TAC levels were observed. TQ treatment (10 mg/kg for 7 days, orally) decreased OSI and TOS but increased TAC (152). The neuroprotective effects of TQ have also been revealed in ischemia-reperfusion spinal cord injury by establishing an oxidant/anti-oxidant balance. In I/R injury rats, there was an increase in oxidative products (MDA and NO) and a decrease in anti-oxidant activities (SOD, CAT, and GSH-Px), which changes TQ treatment (10 mg/kg for 7 days, IP) corrected (153). The TQ neuroprotective effects on LPS-induced brain oxidative stress status in rats have been identified through anti-oxidant and anti-inflammatory pathways. LPS-treated rats in both hippocampus and cortex showed increased levels of MDA and NO but decreased thiol content, SOD, and CAT activities. TQ treatment (2, 5, and 10 mg/kg for 2 weeks, IP) decreased MDA and NO metabolites and increased thiol content, SOD, and CAT activities in LPS-treated rats, which was more pronounced at higher concentrations (154). In the cortex of acrylamide-treated rats, elevated levels of MDA but decreased GSH were evident, and treatment with TQ (2.5, 5, and 10 mg/kg for 11 days, IP) decreased MDA and increased GSH levels (155). TQ (0.5, 2, 4, and 8 mg/kg/ml, IP) improved GSH and SOD levels in valproic acid-induced oxidative stress in perinatal rat brains but no significant difference in MDA levels was found between groups (156). In a rat model of amikacin-induced oxidative damage in the brain tissue, TQ administration inhibited lipid peroxide formation and blocked oxidative reactions, indicated by reduced MDA levels and increased SOD and CAT activities (157).

In various central and peripheral nervous systems disorders induced by head trauma, MCAO, POBCA, pentylenetetrazol, carbon tetrachloride, FA, LPS, arsenic, acrylamide, D-gal, cisplatin, lead, sodium nitrite, radiation, or cerebral vasospasm, extracts or essential oil of *N. sativa* and TQ modulated the pro-oxidant/anti-oxidant balance by reducing and increasing oxidant and anti-oxidant markers, respectively. The anti-oxidant effects of *N. sativa* and TQ on central and peripheral nervous system disorders are summarized in Table 5.

**Diabetes and obesity conditions**

***The effects of various extracts and essential oils of N. sativa***

*In vivo*

The anti-oxidant effects of *N. sativa* seed polysaccharides (NSSP) on type 2 diabetic mice have been shown. In type 2 diabetic rats, elevated MDA levels and decreased TAC, SOD, and CAT occurred, and NSSP treatment (35, 70, and 140 mg/kg for 4 weeks, orally) improved oxidant/anti-oxidant balance (158). Increased levels of MDA and TOS but decreased TAC and SOD have also been reported in diabetic female rats. *N. sativa* oil treatment (0.2 mg/kg for 24 days, orally) decreased MDA and TOS levels but increased serum levels of TAC and SOD (159). The anti-oxidant effects of *N. sativa* fixed and essential oils have also been investigated in streptozotocin (STZ)-induced diabetes mice. Although *N. sativa* fixed and essential oils had no effect on SOD and CAT levels in STZ-treated rats, both oils corrected reduced levels of GSH-Px, GR, and GST (160). The anti-oxidant effects of hydroalcoholic extract of *N. sativa* seed in the hippocampus of STZ-induced diabetic rats have been demonstrated. Elevated hippocampus MDA and decreased thiol levels in STZ-treated rats were prevented by hydroalcoholic extract of *N. sativa* seed treatment (200 and 400 mg/kg for 42 days, orally) (161). Increased MDA and decreased SOD levels in the pancreatic tissue of STZ-treated rats have also been reported. Treatment with *N. sativa* aqueous (2 ml/kg for 30 days, IP) and oil (0.2 ml/kg for 30 days, IP) modified changes in a duration-dependent behavior (162). Another study also found increased levels of MDA and decreased TAC in STZ-treated rats. Treatment with *N. sativa* fixed (4% for 56 days, orally) and essential oil (0.3% for 56 days, orally) prevented oxidative/anti-oxidant changes (163). In STZ-treated rats, serum and cardiac tissue levels of MDA and NO were elevated while GSH, GST, and CAT were decreased. Treatment with *N. sativa* oil (1 ml/kg for 14 days, orally) significantly prevented oxidant/anti-oxidant changes (164). In addition, in STZ-treated rats, pancreatic tissue SOD and MDA alterations were improved by treatment with aqueous (2 ml/kg for 30 days, IP) and oil (0.2 ml/kg for 30 days, IP) of *N. sativa* (58, 165). Also, the use of *N. sativa* extract (200 mg/kg for 21 days, IP) in STZ-treated rats has prevented increased oxidants (MDA and NO) and decreased anti-oxidants markers (SOD and GSH) (166). *N. sativa* anti-oxidant effects (1%/food for 7 weeks, orally) have also been reported in STZ-treated rats with decreased MDA and increased TAC, SOD, and GSH-Px levels (167). *N. sativa* oil protective effects (2 ml/kg for 30 days, orally) against the reduction of pancreatic and hepatic tissues anti-oxidant markers (GSH and CAT) have also been identified (168).

Oxidant/anti-oxidant imbalance was observed in erythrocytes and liver of hyperlipidemic-treated rats as lipid hydroperoxide, MDA, and GSSG increased and SOD, CAT, GST, GSH-Px, total-sh, free-sh, and pro-sh decreased. Pretreatment with methanolic extract (ME) (500 mg/kg for 30 days, orally) or VO fractionated *N. sativa* (100 mg/kg for 30 days, orally), improved this oxidant/anti-oxidant status (169). Hypercholesterolemic-induced oxidative stress has been revealed in rabbits with decreased TAC, and *N. sativa* seed essential oil treatment (5% for 8 weeks, orally) improved it (170). In the high-fat diet-treated rats, an increase in MDA level but a decrease in TAC and CAT have been reported. Treatment with hydro-alcoholic extract of *N. sativa* (200 mg/kg for 35 days, orally) decreased MDA and increased CAT and TAC (171).

*Clinical*

In obese/overweight women (n = 39) *N. sativa* oil supplementation at a dose of 2000 mg/day significantly increased serum TAC and reduced serum MDA (172). *N. sativa* oil concurrent with a low-calorie diet increased SOD levels in obese women (n = 25) but no significant changes in lipid peroxidation, GSH-Px, and TAC levels were observed (173). In hemodialysis patients (n = 25), consumption of *N. sativa* oil as a supplement, decreased MDA level compared to the placebo (174). In patients with diabetes mellitus undergoing hemodialysis (n = 23), *N. sativa* oil supplementation significantly decreased MDA levels and enhanced SOD and TAC levels (175).

A clinical study revealed favorable effects for *N. sativa* oil extract in reducing MDA and NO among patients (n = 23) with type 2 diabetes mellitus (T2DM) (176). Long-term supplementation with *N. sativa* decreased TBARS and elevated TAC, SOD, and glutathione in T2DM patients treated with oral hypoglycemic drugs (177). *N. sativa* consumption in newly diagnosed T2DM patients increased TAC comparable to metformin in this regard (178).

***Effects of TQ***

*In vivo*

TQ protective effects (35 mg/kg for 5 weeks, orally) on pancreatic tissue have also been observed in STZ-treated rats by decreasing TBARS expression levels and increasing GSH and SOD expression levels (179). In addition, TQ (50 mg/kg for 30 days, IP) has been shown to have protective effects in STZ-treated rats by decreasing MDA levels and increasing SOD activity (180). In STZ-treated rats, increased MDA and NO levels but decreased TAC were modified by TQ treatment (50 mg/kg for 4 weeks, orally) (181). TQ treatment (50 mg/kg for 12 weeks, orally) in STZ-treated rats prevented cardiomyopathy by reducing plasma and heart tissue MDA levels but increasing SOD activity with the possible mechanism of increasing Nrf2 expression (52). Decreased anti-oxidant activity of CAT, GSH-Px, GST, and levels of low-molecular-weight anti-oxidants, vitamin C and vitamin E, and GSH were observed in STZ-treated rats, while TBARS and hydroperoxides (lipid peroxidation markers) increased. Treatment with TQ (80 mg/kg for 45 days, orally) reversed the oxidative damage observed in diabetic rats (182). In STZ-treated rats increased levels of TBARS but decreased GSH and activities of anti-oxidant enzymes (GSH-Px, GR, and CAT) occurred which were reversed by treatment with TQ (20 mg/kg for 21 days, orally) (183). Increased MDA and decreased SOD levels in the pancreatic tissue of STZ-treated rats were improved by treatment with TQ (3 mg/kg for 30 days, IP) in a duration-dependent behavior (162). In STZ-treated rats, serum and cardiac tissue levels of MDA and NO were elevated while GSH, GST, and CAT were decreased which were prevented by treatment with TQ (10 mg/kg for 14 days, orally) (164). In STZ-treated rats, serum SOD and pancreatic tissue MDA changes were improved with TQ (3 mg/ml for 30 days, IP) (165).

In hypercholesterolemic-treated rats, oxidant/anti-oxidant imbalance as a decrease in CAT, SOD1, and GSH-Px were improved by treatment with TQ-rich fraction (TQRF) (0.5, 1, and 1.5 g/kg for 8 weeks, orally) and TQ (20, 50, and 100 mg/kg for 8 weeks, orally) (57).

Various extracts or essential oils of *N. sativa* and TQ showed protective and therapeutic effects on STZ-induced diabetes and hyperlipidemia-induced oxidative damage by modulating the oxidative/anti-oxidant pathways. The anti-oxidant effects of *N. sativa* and TQ on diabetes and obese conditions were summarized in Table 6.

***Reproductive system disorders***

*In vivo*

Decreased levels of NO but increased levels of LPO, GST, SOD, and TAC in testicular tissue were detected in monosodium glutamate (MSG) challenged rats, which were reversed by *N. sativa* L. seeds (30 g/kg for 21 days, orally) treatment (184). Cadmium-induced testis oxidative damage in rats was demonstrated by increased MDA and decreased SOD, CAT, and GSH levels which were improved by treatment with *N. sativa* (300 and 600 mg/kg for 14 days, orally) (185). I/R-induced ovarian injury in rats increased MDA, and MPO activity but decreased GSH levels and SOD activity which was reversed by treatment with *N. sativa* ethanol extract (500 mg/kg, orally) before I/R-induction (186). In a rat model of testicular degeneration induced with azathioprine, *N. sativa* oil (500 mg/kg) supplementation significantly normalized redox status, and *N. sativa* floral honey (1.4 mL/kg) improved MDA and SOD activity (187). In a rat model of testicular damage induced by bisphenol-A, administration of *N. sativa* oil (5 ml/kg, oral gavage, for 30 days) increased testicular GSH and SOD levels but reduced MDA levels (188). In another study, *N. sativa* oil protective effects on I/R-induced ovary oxidative injury in rats were revealed by the effects on oxidant/anti-oxidant factors such as TAS, TOS, ceruloplasmin, CAT, myeloperoxidase, native thiols, and total thiols (189). The protective effects of *N. sativa* hydro-alcoholic extract on polycystic ovary syndrome (PCOS) mice oocytes have been revealed by modulating oxidant/anti-oxidant responses. *In vitro* study in PCOS mice, showed increased ROS but decreased GSH and GSH-Px1 levels, and treatment with *N. sativa* (0, 1, 50, and 100 µM), improved oxidative/anti-oxidant markers, especially at 50 µM concentrations (190).

*Clinical*

A randomized, triple-blind clinical trial showed that* N. sativa* oil consumption for 6 weeks has no significant effect on the serum levels of oxidative stress markers in postmenopausal women (191).

***Effects of TQ***

*In vivo*

TQ protective effects on valproic acid (VPA)-induced testicular toxicity have been reported in rats by modulating oxidative stress responses. In VPA-treated rats, decreased TAC and increased TOS and OSI levels, were corrected by TQ treatment (50 mg/kg for 14 days, orally) (192). In lead-induced testicular oxidative damage in rats, increased oxidants (MDA and NO) and decreased anti-oxidants (SOD and GSH) were improved by TQ treatment (5 mg/kg for 56 days, orally) (193). In diabetes-induced testicular damage in rats increased MDA and NO but decreased SOD and GSH levels were improved with TQ (50 mg/kg for 12 weeks, orally) (194). In addition, doxorubicin-induced testicular injury in rats showed elevated TOS and decreased TAC levels, and TQ treatment (10 mg/kg for 7 days, IP) increased TAC and decreased TOS in testicular tissue (195). Elevated levels of MDA and decreased TAS (SOD, CAT, GSH-Px, and nitrite/nitrate) in testicular tissue were also seen in I/R-induced testicular injury in rats, and TQ pre-treatment (20 mg/kg, IP) significantly improved them (196). 

To investigate if the administration of TQ to rats with PCOS induced with letrozole changes the expression of three main anti-oxidant enzymes in ovarian tissue the transcripts of GPx-1, SOD-1, and CAT genes in PCOS and TQ treated (5 and 10 mg/kg) groups were assessed. Transcript levels of the GPx-1 gene were significantly decreased in the ovaries of the PCOS group but 5 mg/kg TQ increased the level of GPx-1 gene expression at the level of the control group. The result of real-time RT-PCR indicated that the relative SOD-1 mRNA expression showed no significant change among groups. The level of gene expression of catalase was decreased in the 10 mg/kg TQ group when compared to the control group (197). In the PCOS mice model, the TQ protective effects in *in vitro* maturation (IVM) of oocytes have been revealed through oxidant/anti-oxidant balance. In PCOS-treated rats, elevated ROS and decreased GSH levels were corrected by TQ treatment (0, 1, 10, 100 µM) (198). In I/R-induced ovarian injury in rats, MDA levels increased and GSH-Px and CAT activities decreased. Treatment with TQ (20 and 40 mg/kg, IP) before and after I/R induction decreased MDA level and increased GSH-Px and CAT activities (199).

In summary, it has been shown that extracts or essential oils of *N. sativa* and TQ have therapeutic effects against oxidative damage by reducing oxidants and increasing anti-oxidants in testis damage induced by I/R, monosodium glutamate, cadmium, valproic acid, doxorubicin, lead or diabetes, as well as I/R-induced ovarian injury. The anti-oxidant effects of *N. sativa* and TQ on reproductive system disorders are summarized in Table 7. 

**Table 1:** A summary of the effects of various extracts, essential oil of *Nigella sativa*, and TQ in lung disorders

**Preparations**	**Study design**	**Dose**	**Effects**	**Ref.**
Hydroethanolic Ext	LPS-induced lung inflammation in rats	100, 200, and 400 mg/kg, IP, 14 days	↓MDA ↑total thiol, CAT, and SOD	(22)
Methanolic Ext	BLC-induced rats' pulmonary fibrosis	500 mg/kg/day, IP, 14 days	↓LPO ↑CAT	(23)
Ethanolic Ext	CLP-induced sepsis in rats	125, 250, and 500 mg/kg, orally, 1 dose	↓LPO and MPO ↑GSH	(26)
*N. sativa* Ext	LPS-induced lung inflammation in rats	EO,5 ml/kg, orally, 3 days NS, 500 mg/kg, p.o., 3 days	↓MDA ↑SOD and CAT	(25)
*N. sativa* EO	LPS-induced lung inflammation in rats	EO,5 ml/kg, orally, 3 days	↓MDA ↑SOD and CAT	(25)
	Ovalbumin sensitization in rats	4 ml/kg/day, orally, 31 days	↓MDA and PC ↑GSH, GSH-Px, CAT, and SOD	(21)
	Nicotine-induced lung injury in rats	0.1 g/kg, orally, 18 weeks	↓MDA ↑SOD, CAT, and GSH	(24)
TQ	HBEC-exposed to CS extract	20 and 50 µM	↑SOD, CAT, GSH, and GR	(27)
	Benzo (a) pyrene-exposed A549 NSCLCC	5 µM, for 48 hr incubation	↓MDA, GSH, and TOS	(28)
	A549 adenocarcinoma lung cancer cell line	20, 40 and 60 μM, for 24 hr incubation	↑TOS ↓TAC in A549 cells	(29)
	CP-induced lung oxidative damage in rats	100 mg/kg/day, 14 days	↓TBARS and SOD ↑GSH	(30)
	BLC-induced oxidative stress in rats	5 mg/kg/day, IP, 5 weeks	↓LPO ↑GSH and SOD	(31)
	BLC-induced rat pulmonary fibrosis	10 and 20 mg/kg, p.o., 28 days	↓MDA ↑GSH, SOD, Nrf2 and HO-1	(32)
	PM2.5-induced lung injury in rats	20 and 40 mg/kg, i.g., 14 days	↓MDA ↑GSH-Px, SOD, Nrf2 and HO-1	(33)
	Benzo (a) pyrene (B(a)P)-induced lung injury in rats	50 mg/kg, orally, 8 weeks	↓MDA ↑SOD, GSH-Px, CAT, TAC	(34)
	CLP-induced sepsis in rats	1 mg/kg/day, IP, 3 days	↓MDA ↑GSH	(35)
	HO-induced lung injury in rats		↓LOOH and SH levels	(36)
	Paraquat-induced lung fibrosis in rats	20 and 40 mg/kg, orally, 28 days	↓LPO ↑SOD and CAT	(37)

Ext: extract, Ref.: References, EO: essential oil, BLC: bleomycin, CAT: catalase, ELF-GSH: epithelial lining fluid GSH, HO-1: heme oxygenase-1, GSH: glutathione, GSH-Px: glutathione peroxidase, MDA: malondialdehyde, GST: glutathione-S-transferase, GR: glutathione reductase, LOOH: Lipid hydroperoxides, LPO: Lipid peroxidation, MPO: myeloperoxidase, NO: nitric oxide, Nrf2: nuclear factor-erythroid factor 2-related factor 2, PC: protein carbonyl, SH: SH group of protein, SOD: superoxide dismutase, TAC: total antioxidant capacity, TBARS: thiobarbituric acid reactive substances, TOS: total oxidant status, TQ: thymoquinone, CLP: cecal ligation and puncture, CP: cyclophosphamide, HBEC: human bronchial epithelial cells, CS: cigarette smoke, NSCLCC: non-small cell lung cancer cells, HO: hyperbaric oxygen

**Table 2:** A summary of the effects of various extracts, essential oil of *Nigella sativa*, and TQ in cardiovascular disorders

**Preparations**	**Study design**	**Dose**	**Effects**	**Ref.**
Macerated and methanolic Ext	Doxorubicin-induced cardiotoxicity on H9c2 cells	50 µg/ml	↓ROS production	(38)
Hydroalcoholic Ext	LPS-induced myocardial fibrosis	100, 200, and 400 mg/kg/day, orally, 2 weeks	↓MDA ↑total thiol, SOD, and CAT	(44)
Macerated Ext	I/R-induced heart damage in rats	800 mg/kg, orally, 12 weeks	↓MDA ↑NAD+	(40)
Hydroalcoholic Ext	I/R-induced heart damage in rats	0.005, 0.02, 0.04, and 0.08 mg/ml, perfused hearts for 10 min	↓TBARS and 4-HNE ↑total thiol, SOD, and CAT	(41)
Methanolic Ext and VO	HL-induced lipid peroxidation in rats	ME (100 mg), VO (20 mg), orally, 30 days	↓MDA	(42)
*N. sativa* EO	Hypertension-induced oxidative stress in rats	0.2 ml/kg, IP, 3 weeks	↓MDA ↑GSH	(45)
	L-NAME-induced hypertension in rats	2.5 mg/kg/day, orally, 8 weeks	↓MDA and NADPH oxidase ↑HO-1 activity	(43)
	Lead-induced cardiotoxicity in rats	4 ml/kg orally before lead administration	↓MDA ↑GSH, SOD, and GSH-Px	(39)
*N. sativa* oil	RDBPCT in hypertensive patients	2.5 ml, twice daily, 8 weeks	↓MDA	(46)
	RDBPCT in CRD patients	2 g/daily, 8 weeks	↓MDA ↑TAC	(47)
TQ	Doxorubicin-induced cardiotoxicity in mice	10 and 20 mg/kg, p.o., 14 days	↓LPO ↑GSH, CAT, SOD, GSH-Px, GR, and GST	(48)
	Doxorubicin‐cardiotoxicity in rats	10 mg/kg, IP, 7 days	↓TOS ↑TAS	(49)
	HL-induced lipid peroxidation in rats	10 mg, orally, 30 days		(42)
	Morphine-induced heart oxidative damage in mice	9 and 18 mg/kg, IP, 21 day	↓NO ↑TAC	(50)
	Diazinon-induced heart oxidative damage in rats	2.5, 5, and 10 mg/kg, i.g., 28 days	↓MDA ↑GSH, SOD, CAT, GSH-Px, and GST	(51)
	Diabetes-induced cardiac myopathy in rats	50 mg/kg, by gavage, 12 weeks	↓MDA ↑SOD and Nrf2	(52)
	Isoproterenol-induced MI in rats	20 and 50 mg/kg, orally, 21 days	↓MDA ↑SOD and GSH	(53)
	Prilocaine-induced cardiotoxicity in rats	15 mg/kg, by gavage, 3 days	↓ROS/RNS ↑TAC	(54)
	LPS-induced MPF in mice	2, 5, and 10 mg/kg, IP, 3 weeks	↓MDA ↑Thiol, SOD, and CAT	(55)
	I/R-induced heart damage in rats	Perfused heart, 5 min with 2.5, 5, 10 μmol/L	↓MDA and H2O2 ↑SOD	(56)
	HC model in rats	20, 50, and 100 mg/kg, orally, 8 weeks	↑SOD, CAT, and GSH-Px	(57)
	HC model in rats	0.5, 1, and 1.5 g/kg, orally, 8 weeks	↑SOD1, CAT, and GSH-Px	(57)
	HL model in rats	20 mg/kg, orally, 30 days	↓MDA	(59)
	HC model induced in rabbits	10 and 20 mg/kg, 8 weeks	↓MDA and PC	(60)
	Cholesterol-fed induced atherosclerosis in rabbits	3.5 mg/day, orally, 4 weeks	↓TBARS ↑GSH	(61)
	Cyclosporine and HL-induced atherosclerosis in rabbits	10 mg/kg, orally, 8 weeks	↓MDA and PC	(62)
	I/R-induced heart damage in rats	20 mg/kg, IP	↓TOS and OSI ↑TAC	(63)

Ext: extract, Ref.: References, EO: essential oil, VO: volatile oil, CAT: catalase, GSH: glutathione, GSH-Px: glutathione peroxidase, 4-HNE: 4-hydroxynonenal, GST: glutathione-S-transferase, GR: glutathione reductase, HO-1: heme oxygenase-1, LPO: lipid peroxidation, MDA: malondialdehyde, MPO: myeloperoxidase, NAD: nicotinamide adenine dinucleotide, NADPH: nicotinamide adenine dinucleotide phosphate, NO: nitric oxide, Nrf2: nuclear factor-erythroid factor 2-related factor 2, OSI: oxidative stability index , PC: protein carbonyls, ROS: reactive oxygen species, SH: total thiol groups, SOD: superoxide dismutase, TBARS: thiobarbituric acid reactive substances, TAS: total antioxidant status, TOS: total oxidant status, MI: myocardial injury, TQ: thymoquinone, LP: lipid peroxidation, HL: hyperlipidemia, HC: hypercholesterolemic, MPF: myocardial and perivascular fibrosis, ME: macerated extract, RDBPCT: randomized, double-blind, placebo-controlled trial, TAC: total antioxidant capacity, CAD:coronary artery diseases

**Table 3:** Anti-oxidant effects of *Nigella sativa* and its main constituent, TQ, in gastrointestinal and liver disorders

**Preparations**	**Study design**	**Dose**	**Effects**	**Ref.**
*N. sativa* Ext	APAP-induced in TIB-73 cells	25, 50, 75, and 100 µg/mL	↓ROS	(64)
	APAP-induced hepatotoxicity in rats	100–900 mg/kg, orally, 2 weeks	↓MDA ↑SOD and GSH	(64)
*N. sativa* Ext	Lead acetate-induced OLI in rabbit	20 g/kg, orally, 8 weeks	↓MDA ↑GSH, GSH-Px, and GST	(69)
*N. sativa* fixed Ext	potassium bromate-induced oxidative stress in rats	4%, orally, 56 days	↓MDA ↑TAC, GST, GR, GSH-Px, SOD, α-Tocopherol, and γ-Tocopherol	(71)
*N. sativa* VO	I/R-induced intestinal injury in rat	0.2 ml/kg, IP, before induction of I/R	↓TOS, OSI, and MPO ↑TAC and CAT	(74)
*N. sativa* EO	CCl4-induced hepatotoxicity in rats	2 and 4 ml/kg, orally, 28 days	↓MDA and NO ↑GSH, CAT, SOD	(65)
	Ethanol-induced hepatoxicity in rats	2.5 and 5 ml/kg, orally, 3 weeks	↓MDA ↑GSH	(66)
	CP-induced GI toxicity in rats	2 ml/kg, orally, 14 days	↓LPO ↑GSH, SH, SOD, CAT, GSH-Px, GR, GST	(67)
	CsP-induced hepatotoxicity in rats	50 and 200 mg/kg, orally, 15 days	↑GSH, SOD, and CAT	(68)
	AlCl₃-induced oxidative injury in the liver in rats	2 ml/kg, by gavage, 8 weeks	↓MDA ↑GSH, GSH-Px, CAT, and SOD	(70)
	Potassium bromate-induced oxidative stress in rats	3%, orally, 56 days	↓MDA ↑TAC, GST, GR, GSH-Px, SOD, α-Tocopherol, and γ-Tocopherol	(71)
	TiO_2_-induced liver cirrhosis in rats	5 and 10 ml/kg, orally, 8 weeks	↓TBARS ↑CAT, SOD, GSH-Px, and GSH	(72)
	I/R-induced gastric mucosal lesion in rats	2.5 and 5 ml/kg, p.o., before I/R	↓LPO ↑GSH and SOD	(73)
	I/R-induced hepatic injury in rats	0.2 ml/kg, IP, before induction of I/R	↓TOS, MPO, and OSI ↑CAT and TAC	(75)
	Acrylamide-induced liver toxicity in rats	10 mg/kg, IP, 15 days	↓MDA level in liver tissue ↑GSH and SOD activity	(76)
*N. sativa* oil	Arsenic-induced redox imbalance	2 ml/kg, orally., 44 days	↑antioxidant defense enzymes in intestinal mucosal	(77)
*N. sativa*	Dietary frying oil-induced hepatotoxicity in rats	2% *Nigella* + 7% frying oils, 30 days	↓Oxidative stress and hepatic biomarkers	(78)
*N. sativa* powder	RDBPCT in patients with ulcerative colitis	2 g/day, 6 weeks	↓MDA	(79)
TQ	Acrylamide-induced OLI in rats	10 and 20 mg/kg, orally, 21 days	↓NO and MDA ↑GSH, SOD, GSH-Px, and CAT	(81)
	Arsenic-induced hepatotoxicity in rats	10 mg/kg, orally, 28 days	↓MDA ↑CAT, SOD, GR, GSH-Px, and GSH	(82)
	Gentamicin-induced liver injury in rats	20 mg/kg, orally, 21 days	↓MDA ↑GSH, GSH-Px, and SOD	(83)
	Bisphenol A-induced hepatotoxicity in rats	10 mg/kg, orally, 5 weeks	↓MDA and LPO ↑TAC, GSH, GSH-PX, SOD, GST, CAT	(84)
	ATO-induced hepatic injury in rats	30 mg/kg, orally, 10 consecutive days	↓MDA and PC ↑GSH and CAT	(85)
	TCDD-induced hepatotoxicity in rats	50 mg/kg, orally, 30 days	↓MDA and TOS ↑GSH, CAT, SOD, and TAC	(86)
	CCl4-induced liver injury in mice	10 mg/kg, orally, 12 weeks	↑TAC, SOD, GSH-Px, and CAT	(87)
	Diazinon-induced hepatic injury in rats	40 mg/kg, orally, 21 days	↓ROS, NO, H_2_O_2_, MDA and protein carbonyl ↑TAC, SOD, CAT, GSH, GPX, and GST	(88)
	Paraquat-induced hepatoxicity in mice	5, 10, and 20 mg/kg, orally, 4 days	↓LPO ↑TAC and SOD	(89)
	NAFLD model in rats	10 and 20 mg/kg, by gavage, 6 weeks	↓MDA ↑TAC	(90)
	LPS-induced liver fibrosis in rats	2, 5, and 10 mg/kg, IP, 3 weeks	↓MDA ↑CAT and SOD	(91)
	Partial hepatectomy (PH) under I/R in rats	30 mg/kg, orally, 10 days	↓MDA and CD ↑GSH-Px, SOD, CAT, and SP	(92)
	I/R intestinal injury in rats	50 mg/kg, IP, before ischemia	↓SOD, GSH, and MDA	(93)
	Partial hepatic warm I/R in rats	30 mg/kg, orally, 10 days	↓MDA and CD ↑sulfhydryl proteins	(94)
	I/R-induced gastric mucosal injury in rats	10 and 30 mg/kg before I/R induction	↓MDA and MPO ↑SOD, GSH, and NO	(95)
	I/R-induced intestinal injury in rats	50 mg/kg, orally	↓MDA and MPO ↑GSH	(96)
	I/R-induced hepatic injury in rats	5, 20, and 50 mg/kg, orally, 10 days	↓MDA ↑GSH	(97)
	I/R-induced hepatic injury in rats	20 mg/kg, orally, 10 days	↓MDA ↑GSH	(98)
	CsA-induced hepatotoxicity in rats	10 mg/kg, by gavage, 28 days	↓MDA ↑GSH and SOD	(99)
	I/R-induced acute liver injury in rats	10 mg/kg, by gavage 1 day	↓MDA ↑GSH and SOD	(99)
	Cadmium-induced OLI in mice	10 µM, IP	↓PC ↑GSH	(80)
	CP-induced hepatotoxicity in rats	500 mg/kg, orally, 30 days	↓MDA ↑GSH, GST, CAT, SOD, and GSH-Px	(100)
	Lead-induced OLI in rats	5 mg/kg, orally, 5 weeks	↑SOD, GSH-Px, CAT, GR, and GSH	(101)
	APAP-hepatotoxicity in rats	5 mg/kg, orally, 24 hr	↓GSSG and MDA ↑SOD and GSH-Px	(102)
	Cholestatic-induced liver injury in rats	25 and 50 mg/kg, by gavage, 15 days	↓HP and MDA ↑SOD and GSH-Px	(103)
	Methotrexate-induced liver toxicity in rats	10 mg/kg, orally, 10 days	↓NO and MDA ↑CAT and GSH	(104)
	Tamoxifen-induced LI in female rats	50 mg/kg, orally, 20 days	↓LPO ↑SOD and CAT	(105)
	Aflatoxin B1 induced liver toxicity in mice	4.5, 9, and 18 mg/kg, orally, 3 days	↓MDA ↑GSH	(106)
	CsP-induced hepatotoxicity in mice	5 and 10 mg/kg, IP, 3 days	↑SOD and CAT	(107)
	HC-induced hepatotoxicity in mice	5 and 10 mg/kg, IP, 3 days	↑SOD and CAT	(107)
	Tuberculosis drugs-induced LD in rats	10, 20, and 40 mg/kg, orally, 8 weeks	↓LPO ↑SOD and CAT	(108)
	APAP-induced OLI in mice	0.5, 1, and 2 mg/kg, orally, 5 days	↓LPO ↑GSH	(109)
	TiO_2_ nanoparticles-induced TLI	20 mg/kg, orally, 6 weeks	↓LPO ↑GSH and TBARS	(110)
	THI-induced oxidative stress in rats	50 mg/kg, IP, 30 days	↓TOS, LPO, and OSI ↑TAS, sulfhydryl, and PON	(111)
	I/R-induced liver injury in rats	20 mg/kg, orally, 10 days	↓MDA, MPO, and NO ↑GSH	(98)
	I/R-induced gastric mucosal lesion in rats	5, 20, 50 and 100 mg/kg, p.o. before I/R	↓LPO ↑GSH and SOD	(73)

Ext: extract, Ref.: References, EO: essential oil, VO: volatile oil, GI: gastrointestinal, APAP: acetaminophen, ATO: atorvastatin, CCl4: carbon tetrachloride, CAT: catalase, CP: cisplatin, CsA: cyclosporine A; CsP: cyclophosphamide, GSH: glutathione, GSH-Px: glutathione peroxidase, GST: glutathione-S-transferase, GR: glutathione reductase, HO-1: heme oxygenase-1, HP: hydroxyproline, I/R: ischemic/reperfusion, LPO: lipid peroxide, LPS: lipopolysaccharide, MDA: malondialdehyde, MPO: myeloperoxidase, NAFLD: nonalcoholic fatty liver disease, NO: nitric oxide, Nrf2: Nuclear factor-erythroid factor 2-related factor 2, OSI: oxidative stability index , PC: protein carbonyl, PON: paraoxonase, ROS: reactive oxygen species, SH: total thiol groups, SOD: superoxide dismutase, TBARS: thiobarbituric acid reactive substances, TAS: total antioxidant status, TCDD: tetrachlorodibenzo-p-dioxin, TOS: total oxidant status, TQ: thymoquinone, OLI: oxidative liver injury, AlCl₃: aluminum chloride, SP: sulfhydryl proteins, CD: conjugated dienes, HC: hyperbilirubinemia and cyclophosphamide. THI: total head irradiation, TiO2: titanium dioxide, RDBPCT: randomized, double-blind, placebo-controlled trial, TAC: total antioxidant capacity

**Table 4:** Anti-oxidant effects of *Nigella sativa* and its main constituent, TQ, in renal disorders

**Preparations**	**Study design**	**Dose**	**Effects**	**Ref.**
Hydroalcoholic Ext	Diabetes-induced kidney OD in rats	200 and 400 mg/kg, orally, 6 weeks	↓MDA ↑Thiol, SOD, and CAT	(112)
	Cisplatin-induced nephrotoxicity in rat	0.5 g/kg, by gavage, 60 days	↓MDA ↑CAT, GSH-Px, and SOD	(113)
	I/R-induced renal injury in rat	150 and 300 mg/kg, i.v.	↓MDA ↑total thiol	(117)
	I/R-induced renal injury in rat	150 and 300 mg/kg, i.v.	↓MDA ↑total thiol	(117)
	PTU-induced renal OD and hypothyroidism in rats	100, 200, and 400 mg/kg, orally, 8 weeks	↓MDA and total thiols ↑SOD and CAT	(118)
*N. sativa *powder	Cisplatin-induced nephrotoxicity in rats	3g/kg, by gavage, 60 days	↓MDA ↑CAT, GSH-Px, and SOD	(113)
Macerated Ext	I/R-induced renal injury in rats	0.5, 1, and 2%, orally, 3 weeks	↓MDA ↑SOD, CAT, GSH, and GSH-Px	(114)
*N. sativa* VO	I/R-induced renal injury in rat	0.2 ml/kg IP, before I/R	↓TOS, MPO, and OSI ↑TAC and CAT	(115)
*N. sativa* EO	Cisplatin-induced nephrotoxicity in rats	2 g/kg, by gavage, 60 days	↓MDA ↑CAT, GSH-Px, and SOD	(113)
	Cisplatin-induced renal OD in rats	2 ml/kg, orally, 14 days	↓MDA ↑GSH and total thiol	(119)
	Radiation-induced renal OD in rats	1 g/kg, by gavage, 10 days	↓ LOPs, TOS, and OSI ↑PON_1_ and ceruloplasmin	(120)
	I/R-induced renal injury in rats	0.3 ml/kg, by gavage, 7 days	↓MDA, PC, and NO ↑SOD and GSH-Px	(116)
	I/R-induced renal injury in rats	0.6 ml/kg, by gavage after I/R	↓MDA, PC, and NO ↑SOD and GSH-Px	(116)
TQ	Radiation-induced renal OD in rats	50 mg/kg, IP, 10 days	↓ LOPs, TOS, and OSI ↑ PON_1_ and ceruloplasmin	(120)
	Cisplatin-induced renal oxidative injury	0.5–3 mg/kg, orally, 14 days	↓MDA ↑GSH and Total-SH	(119)
	Gentamicin-induced renal OD in rats	20 mg/kg, orally, 21 days	↓MDA ↑GSH, SOD, and GSH-Px	(121)
	Cadmium-induced nephrotoxicity in rats	50 mg/kg, orally, 30 days	↓MDA ↑CAT, SOD, and GSH-Px	(122)
	NaNO_2_-induced renal toxicity in rats	25 and 50 mg/kg, orally, 3 months	↓MDA ↑GSH	(123)
	Sodium arsenite-induced nephrotoxicity in rats	10 mg/kg, orally, 28 days	↓MDA and NO ↑GSH, SOD, CAT. GSH-Px, and GR	(124)
	Acrylamide-induced nephrotoxicity in rats	10 and 20 mg/kg, orally, 21 days	↓MDA and NO ↑GSH, GSH, GSH-Px, SOD and CAT	(81)
	Oxonic acid-induced hyperuricemia in rats	10 and 20 mg/kg, orally, 12 weeks	↑CAT, SOD, GR, GSH-Px, and aconitase	(125)
	Diclofenac-induced kidney injury in rats	20 mg/kg, orally, 21 days	↓MDA ↑GSH, TAC, and CAT	(126)
	Gentamicin-induced renal failure in rats	10–30 mg/kg, orally, 28 days	↓MDA ↑GSH, SOD, and GSH-Px	(127)
	Arsenic-induced nephrotoxicity in rat	10 mg/kg, i.g., 15 days	↓MDA ↑SOD, CAT, and GSH-Px	(122)
	C_12_H_4_Cl_4_O-induced nephrotoxicity in rats	50 mg/kg, orally, 30 days	↓MDA and TOS ↑GSH, CAT, SOD, TAS	(129)
	LPS-induced renal fibrosis in rats	2–10 mg/kg, IP, 21 days	↓MDA ↑total thiol, SOD, and CAT	(130)
	Vancomycin-induced nephrotoxicity in rats	10 mg/kg, IP, 8 days	↓MDA ↑SOD and GSH-Px	(131)
	Contrast-induced nephropathy in rats	1 and 1.75 mg/kg, IP, 4 days	↓MDA ↑SOD	(132)
	I/R-induced renal injury in rats	10 mg/kg, orally, 10 days	↓MDA ↑GST	(134)
	Cyclosporine A-induced nephrotoxicity in rats	10 mg/kg, by gavage, 28 days	↓MDA ↑SOD and GSH	(99)
	I/R-induced acute renal injury in rats	10 mg/kg, by gavage, 1 day	↓MDA ↑SOD and GSH	(99)
	PROP-induced hypothyroidism in rats	50 mg/kg, by gavage, 4 weeks	↓MDA ↑GSH-Px, SOD, and CAT	(135)
	Cisplatin-induced renal OD in rats	1.5 mg/kg, 14 days	↓MDA ↑GSH and total thiol	(119)
	Carfilzomib-induced renal impairment	10 and 20 mg/kg, 16 days	↓MDA ↑GSH, SOD, and CAT	(133)

Ext: extract, Ref.: References, EO: essential oil, VO: volatile oil, CAT: catalase, GSH: glutathione, GSH-Px: glutathione peroxidase, GST: glutathione-S-transferase, GR: glutathione reductase, HO-1: heme oxygenase-1, LPO: lipid peroxidation, LPx: lipid peroxide, MDA: malondialdehyde, MPO: myeloperoxidase, NO: nitric oxide, Nrf2: nuclear factor-erythroid factor 2-related factor 2, OSI: oxidative stability index, PC: protein carbonyl, SH: total thiol groups, SOD: superoxide dismutase, TBARS: thiobarbituric acid reactive substances, TAS: total antioxidant status, TOS: total oxidant status, TQ: thymoquinone, OD: oxidative damage, PTU: propylthiouracil, LOPs: lipid hydroperoxide, PON1: paraoxonase. NaNO2: sodium nitrite, C12H4Cl4O: tetrachlorodibenzo-p-dioxin, PROP: propylthiouracil, TAC: total antioxidant capacity

**Table 5:** Anti-oxidant effects of *Nigella sativa* and its main constituent, TQ, in central and peripheral nervous systems disorders

**Preparations**	**Study design**	**Dose**	**Effects**	**Ref.**
Hydroalcoholic Ext	MCAO-induced cerebral ischemia	400 mg/kg, orally, 7 days	↓TBARS ↑GSH, SOD, and CAT	(138)
Chloroform and PE Ext	MCAO-induced cerebral ischemia	400 mg/kg, orally, 7 days	↓TBARS ↑GSH, SOD, and CAT	(139)
*N. sativa *Ext	Experimental head trauma in rats		↓MDA ↑TAS	(140)
Hydroalcoholic Ext	POBCA-induced cerebral hypoperfusion in rats	00, 200, and 400 mg/kg, IP, 10 days	↓MDA ↑SOD	(141)
*N. sativa* EO	Pentylenetetrazol-induced kindling seizures in mice	12 ml/kg, orally, 21 days	↓MDA, XO, and ADA ↑SOD and GSH-Px	(136)
	Carbon tetrachloride-induced hepatotoxicity	2 and 4 ml/kg, orally, 28 days	↓MDA and NO ↑CAT, SOD, and GSH	(65)
	FA-induced sub-acute inflammation	4 ml/kg, orally, 7 days	↓MDA and GSSG ↑GSH, SOD, and DH	(137)
	Radiation-induced OS in rat brain	1 g/kg, orally, 10 days	↓XO, TOS, OSI, and LOOH ↑TSSA, GST, GSH-Px, NSSA, SOD, and PON, TAS, and total thiol	(146)
	SGD-induced cell death in cultured PC12 cells	15.62–250 µg/ml for 2 hr	↓ROS	(142)
TQ	SGD-induced cell death in cultured PC12 cells	1.17–150 µM for 2 hr	↓ROS	(142)
	LPS-induced NI in BV2 mouse microglia cell line	2.5–10 µM for 12 hr	↑Nrf2, HO-1, ARE, and NQO1	(143)
	LPS/IFNγ or H_2_O_2_-activated BV-2 microglia	0-40 µM	↓MDA, superoxide, NO, hydrogen peroxide, and LPO ↑GSH, SOD, and CAT	(145)
	Arsenic-induced neurotoxicity in rats	10 mg/kg, IP, 21 days	↓MDA and NO ↑GR, SOD, GSH, CAT, and GSH-Px	(147)
	Animal model of Parkinson's diseases	7.5 and 15 mg/kg, p.o.	↓PAB	(148)
	Acrylamide-induced brain oxidative damage in rats	0 and 20 mg/kg, p.o., 21 days	↓MDA and NO ↑GSH, GSH-Px, SOD, CAT	(81)
	D-gal-induced memory impairments in rats	2.5, 5, 10 mg/kg, IP, 8 weeks	↓MDA ↑GSH	(149)
	Cisplatin-induced neurotoxicity in rats	20 mg/kg, p.o. three times a week	↓MPO and 8-isoprostane ↑SOD	(150)
	Lead-induced brain oxidative damage in rats	10 mg/kg, p.o., 20 days	↓MDA ↑GSH-Px, SOD, and CAT	(151)
	SN-induced brain oxidative damage in rats	50 mg/kg, p.o., 12 weeks	↓MDA ↑GSH and Nrf2	(144)
	CVS-induced SH in rats	10 mg/kg, p.o., 7 days	↓TOS and OSI ↑TAC	(152)
	I/R-induced spinal cord injury in rats	10 mg/kg, IP, 7 days	↓MDA and NO ↑SOD, CAT, and GSH-Px	(153)
	LPS-induced brain OS in rats	2, 5, and 10 mg/kg, IP, 2 weeks	↓MDA and NO ↑thiol content, SOD, and CAT	(154)
	Acrylamide-induced CNS toxicity in rats	2.5, 5, 10 mg/kg, IP, 11 days	↓MDA ↑GSH	(155)
	POBCA-induced cerebral hypoperfusion in rats	10, 20, 40 mg/kg, IP, 10 days	↓MDA ↑SOD	(141)
	Radiation-induced OS in rat brain	50 mg/kg, IP, 10 days	↓XO, TOS, OSI, and LOOH ↑TSSA, GST, GSH-Px, NSSA, SOD, PON, TAS, thiol	(146)
	Valproic acid-induced oxidative stress in rat brain	0.5, 2, 4, and 8 mg/kg/ml, IP	↑GSH and SOD	(156)
	Amikacin-induced oxidative damage	40 mg/kg, p.o.	↓MDA ↑SOD and CAT	(157)

Ext: extract, Ref.: References, EO: essential oil, PE: petroleum ether, ADA: adenosine deaminase, CAT: catalase, DH: hydrogen donor capacity, GSH: glutathione, GSH-Px: glutathione peroxidase, GST: glutathione-S-transferase, GR: glutathione reductase, GSSG: glutathione disulfide, HO-1: heme oxygenase-1, LOOH: lipid hydroperoxide, LPO: lipid peroxidation, LPx: lipid peroxide, MDA: malondialdehyde, MPO: myeloperoxidase, NO: nitric oxide, NQO1: NAD(P)H quinone dehydrogenase 1, Nrf2: nuclear factor-erythroid factor 2-related factor 2, NSSA: non-enzymatic superoxide scavenger activity, OSI: oxidative stability index, PAB: prooxidant-antioxidant balance, PON: paraoxonase, ROS: reactive oxygen species, SH: total thiol groups, SOD: superoxide dismutase, TBARS: thiobarbituric acid reactive substances, TAS: total antioxidant status, TOS: total oxidant status, TQ: thymoquinone. TSSA: total superoxide scavenger activity, XO: xanthine oxidase, MCAO: middle cerebral artery occlusion, OS: oxidative stress, POBCA: permanent occlusion of bilateral common carotid arteries, FA: Freund's adjuvant, SGD: serum/glucose deprivation, NI: neuroinflammation, D-gal: D-galactose, SN: sodium nitrite, CVS: cerebral vasospasm, SH: subarachnoid hemorrhage, CNS: central nervous system, TAC: total antioxidant capacity

**Table 6:** Antioxidant effects of *Nigella sativa* and its main constituent, TQ, on diabetes and obesity conditions

**Preparations**	**Study design**	**Dose**	**Effects**	**Ref.**
*N. sativa* polysaccharides	STZ-induced diabetic rats	35, 70, and 140 mg/kg, orally, 4 weeks	↓MDA ↑TAC, SOD, and CAT	(158)
*N. sativa* fixed Ext.	STZ-induced diabetic mice		↑GSH-Px, GR, and GST	(160)
Hydroalcoholic Ext	STZ-induced diabetic rats	200 and 400 mg/kg, orally, 42 days	↓MDA ↑thiol	(161)
Aqueous Ext.	STZ-induced diabetic rats	2 ml/kg, IP, 30 days	↓MDA ↑SOD	(162)
*N. sativa* fixed Ext	STZ-induced diabetic rats	4%, orally, 56 days	↓MDA ↑TAC	(163)
Aqueous Ext	STZ-induced diabetic rats	2 ml/kg, IP, 30 days	↓MDA ↑SOD	(165)
*N. sativa* Ext	STZ-induced diabetic rats	200 mg/kg, IP, 21 days	↓MDA and NO ↑SOD and GSH	(166)
*N. sativa* Ext	STZ-induced diabetic rats	1%, orally, 7 weeks	↓MDA ↑TAC, SOD, and GSH-Px	(167)
Methanolic Ext	Hyperlipidemia-induced oxidative damage	500 mg/kg, orally, 30 days	↓LPO, MDA, and GSSG ↑SOD, CAT, GST, GSH-Px, total-SH, free-SH and pro-SH	(169)
*N. sativa* VO	Hyperlipidemia-induced oxidative damage	500 mg/kg, orally, 30 days	↓LPO, MDA, and GSSG ↑SOD, CAT, GST, GSH-Px, total-SH, free-SH and pro-SH	(169)
Hydro-alcoholic Ext	High-fat diet-treated rats	200 mg/kg, orally, 35 days	↓MDA ↑TAC and CAT	(171)
*N. sativa* EO	STZ-induced diabetic rats	2 ml/kg, orally, 30 days	↑GSH and CAT	(168)
	HC-induced OS in rabbit	5%, orally, 8 weeks	↑TAC	(170)
	STZ-induced diabetic rats	0.2 ml/kg, IP, 30 days	↓MDA ↑SOD	(165)
	STZ-induced diabetic rats	0.3%, orally, 56 days	↓MDA ↑TAC	(163)
	STZ-induced diabetic rats	1 ml/kg, orally, 14 days	↓MDA and NO ↑GSH, GST, and CAT	(164)
	STZ-induced diabetic rats	0.2 ml/kg IP, 30 days	↓MDA ↑SOD	(162)
	STZ-induced diabetic mice		↑GSH-Px, GR, and GST	(160)
	STZ-induced diabetic rat	0.2 mg/kg, orally, 24 days	↓MDA and TOS ↑TAC and SOD,	(159)
*N. sativa* oil	Crossover RDBPCT in obese/overweight women	2000 mg/day, 8 weeks	↓MDA ↑TAC	(172)
	RDBPCT in obese women	3 g/day, 8 weeks	↑SOD	(173)
	RDBPCT in hemodialysis patients	1000 mg/day, 8 weeks	↓MDA	(174)
	RDBPCT in DM patients	2 g/day, 12 weeks	↓MDA ↑SOD and TAC	(175)
	RDBPCT in T2D patients	500 mg twice daily, 8 weeks	↓MDA and NO	(176)
*N. sativa*	RDBPCT in patients with T2DM	2 g/day, 1 year	↓TBARS ↑TAC, SOD, and glutathione	(177)
	RDBPCT in T2DM patients	1350 mg/day, 3 months	↑TAC	(178)
TQ	STZ-induced diabetic rats	35 mg/kg, orally, 5 weeks	↓TBARS ↑GSH and SOD	(179)
	STZ-induced diabetic rats	50 mg/kg IP, 30 days	↓MDA ↑SOD	(180)
	STZ-induced diabetic rats	50 mg/kg, orally, 4 weeks	↓MDA and NO ↑TAC	(181)
	STZ-induced diabetic rats	50 mg/kg, orally, 12 weeks	↓MDA ↑SOD and Nrf2	(52)
	STZ-induced diabetic rats	80 mg/kg, orally, 45 days	↓TBARS ↑CAT, GSH-Px, GST, GSH,	(182)
	STZ-induced diabetic rats	20 mg/kg, orally, 21 days	↓TBARS ↑GSH, GSH-Px, GR, and CAT	(183)
	STZ-induced diabetic rats	3 mg/kg, IP, 30 days	↓MDA ↑SOD	(162)
	STZ-induced diabetic rats	10 mg/kg, orally, 14 days	↓MDA and NO ↑GSH, GST, and CAT	(164)
	STZ-induced diabetic rats	3 mg/ml, IP, 30 days	↓MDA ↑SOD	(165)
	HC-induced OS in rat	0.5, 1, and 1.5 g/kg, orally, 8 weeks	↑CAT, SOD1, and GSH-Px	(57)
	HC-induced OS in rat	20, 50, and 100 mg/kg, orally, 8 weeks	↑CAT, SOD1, and GSH-Px	(57)

Ext: extract, Ref.: References, EO: essential oil, VO: volatile oil, CAT: catalase, GSH: glutathione, GSH-Px: glutathione peroxidase, MDA: malondialdehyde, GST: glutathione-S-transferase, GR: glutathione reductase, HO-1: heme oxygenase-1, LPO: lipid peroxidation, LPO: lipid peroxide, MPO: myeloperoxidase, NO: nitric oxide, Nrf2: nuclear factor-erythroid factor 2-related factor 2, OSI: oxidative stability index, ROS: reactive oxygen species, SH: total thiol groups, SOD: superoxide dismutase, STZ: Streptozotocin, TBARS: thiobarbituric acid reactive substances, TAS: total antioxidant status, TOS: total oxidant status, TQ: thymoquinone, HC: hypercholesterolemic, OS: oxidative stress, RDBPCT: randomized, double-blind, placebo-controlled trial, TAC: total antioxidant capacity, T2DM: type 2 diabetes mellitus

**Table 7:** **. **Anti-oxidant effects of *Nigella sativa* and its main constituent, TQ, on reproductive system disorders

**Preparations**	**Study design**	**Dose**	**Effects**	**Ref.**
*N. sativa* Ext	Monosodium glutamate-induced testis damage in rats	30 g/kg, orally, 21 days	↓LPO, GST, and TAC ↑NO and SOD2	(184)
Methanolic Ext	Cadmium-induced testis oxidative damage in rats	300 and 600 mg/kg, orally, 14 days	↓MDA ↑SOD, CAT, and GSH	(185)
Ethanolic Ext	I/R-induced ovarian injury in rats	500 mg/kg, orally before I/R-induction	↓MDA and MPO ↑SOD and GSH	(186)
*N. sativa* EO	Azathioprine-induced testicular degeneration	500 mg/kg	↓MDA ↑SOD	(187)
	Bisphenol A-induced testicular degeneration	5 ml/kg, by gavage, 30 days	↓MDA ↑GSH and SOD	(188)
	I/R-induced ovary oxidative injury in rats	2 ml/kg, IP, 1 hr before I/R induction	No effect	(189)
Hydro-alcoholic Ext	PCOS mice oocytes	0, 1, 50, and 100 µM	↓ROS ↑GSH and GSH-Px1	(190)
TQ	Valproic acid-induced testicular toxicity in rats	50 mg/kg, orally, 14 days	↓TOS and OSI ↑TAC	(192)
	Lead-induced testicular oxidative damage in rats	5 mg/kg, orally, 56 days	↓MDA and NO ↑SOD and GSH	(193)
	Diabetes-induced testicular damage in rats	50 mg/kg, orally, 12 weeks	↓MDA and NO ↑SOD and GSH	(194)
	Doxorubicin-induced testicular injury in rats	10 mg/kg, IP, 7 days	↓TOS ↑TAS	(195)
	I/R-induced testicular injury in rats	20 mg/kg, IP	↓MDA ↑SOD, CAT, GSH-Px, and NIT	(196)
	PCOS mice model	0, 1, 10, 100 µM	↓ROS ↑GSH	(198)
	PCOS rat model	5 and 10 mg/kg	↑GPx-1 transcript levels	(197)
	I/R-induced ovarian injury in rats	20 and 40 mg/kg, IP	↓MDA ↑GSH-Px and CAT	(199)
*N. sativa* oil	Triple-blind RCT in postmenopausal women	1000 mg/day, 8 weeks	no significant effect on MDA and TAC	(191)

Ext: extract, Ref.: References, EO: essential oil, CAT: catalase, GSH: glutathione, GSH-Px: glutathione peroxidase, MDA: malondialdehyde, GST: glutathione-S-transferase, GR: glutathione reductase, HO-1: heme oxygenase-1, LPO: lipid peroxidation, LPx: lipid peroxide, MPO: myeloperoxidase, NIT: nitrite nitrate, NO: nitric oxide, Nrf2: nuclear factor-erythroid factor 2-related factor 2, OSI: oxidative stability index, ROS: reactive oxygen species, SH: total thiol groups, SOD: superoxide dismutase, TBARS: thiobarbituric acid reactive substances, TAS: total antioxidant status, TOS: total oxidant status, TQ: thymoquinone, PCOS: Polycystic ovary syndrome, TAC: total antioxidant capacity, RCT: randomized controlled trial

**Figure 3:**
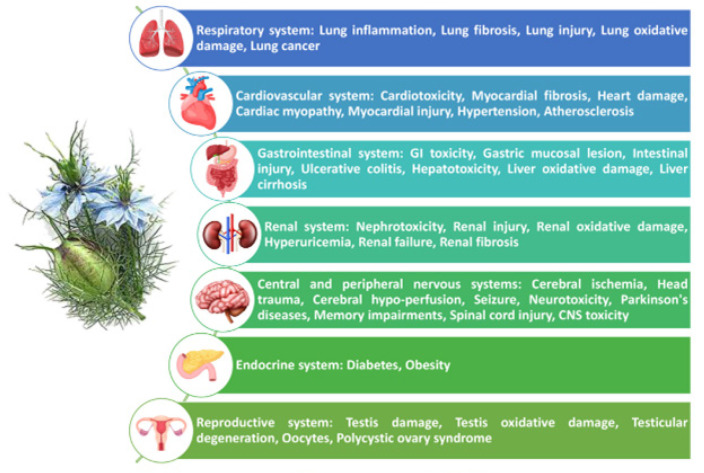
Anti-oxidant effects of *Nigella sativa* and thymoquinone on various body systems

**Figure 4:**
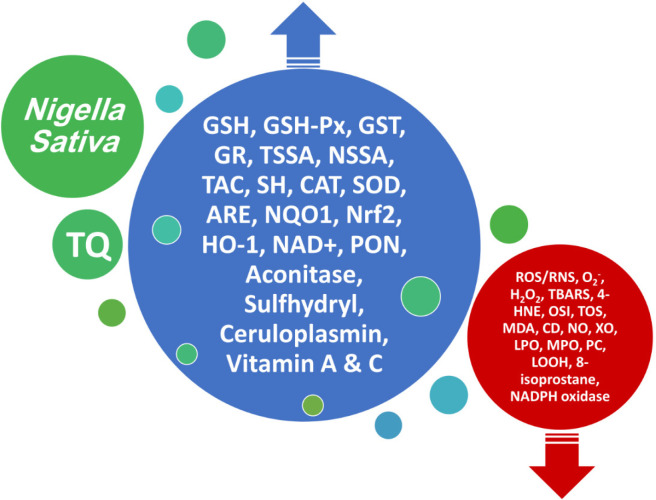
**. **Possible molecular mechanisms of the anti-oxidant effects of *Nigella sativa* and thymoquinone

4-HNE: 4-hydroxynonenal, CAT: catalase, CD: conjugated dienes, GR: glutathione reductase, GSH: glutathione, GSH-Px: glutathione peroxidase, GST: glutathione-S-transferase, HO-1: heme oxygenase-1, LOOH: lipid hydroperoxide, LPO: lipid peroxide, MDA: malondialdehyde, MPO: myeloperoxidase, NAD: nicotinamide adenine dinucleotide, NADPH: nicotinamide adenine dinucleotide phosphate, NO: nitric oxide, NQO1: NAD(P)H quinone dehydrogenase 1, Nrf2: Nuclear factor-erythroid factor 2-related factor 2, NSSA: non-enzymatic superoxide scavenger activity, OSI: oxidative stability index, PC: protein carbonyl, PON: paraoxonase, ROS: reactive oxygen species, SH: total thiol groups, SOD: superoxide dismutase, TAS: total anti-oxidant status, TBARS: thiobarbituric acid reactive substances, TOS: total oxidant status, TQ: thymoquinone, TSSA: total superoxide scavenger activity, XO: xanthine oxidase

## Discussion

The anti-oxidant property of *N. sativa* and TQ on a wide variety of disorders was indicated (Figure 3). *N. sativa* and TQ have been found to exhibit anti-oxidant effects in pulmonary disorders. These effects are mediated through the reduction of oxidants and the augmentation of anti-oxidants. The intricate interplay between oxidant and anti-oxidant molecules is crucial in maintaining the delicate balance required for optimal pulmonary function. Similarly, in various cardiovascular disorders, *N. sativa* and TQ have been shown to modulate the equilibrium between the oxidant and anti-oxidant systems. Notably, TQ exerts its influence on the pro-oxidant and anti-oxidant balance by activating the Nrf2 signaling pathway. This pathway plays a pivotal role in the regulation of cellular anti-oxidant defenses, thereby contributing to the overall protective and therapeutic effects observed in cardiovascular disorders. In gastrointestinal and liver disorders, the anti-oxidant effects of *N. sativa* and TQ have been reported by modulating the oxidative/anti-oxidant pathways in oxidative stress conditions. These disorders are characterized by dysregulation of the oxidative/anti-oxidant equilibrium, which can lead to detrimental consequences for gastrointestinal and hepatic tissues. The administration of *N. sativa* extracts and TQ has shown promise in restoring this balance and ameliorating the associated oxidative damage. Moreover, the plant and TQ have demonstrated protective and therapeutic effects on kidney oxidative stress in various renal disorders. Renal disorders often manifest as a result of imbalances in the oxidative/anti-oxidant pathways, leading to impaired renal function and tissue damage. However, the administration of *N. sativa* extracts and TQ has been shown to exert a protective effect by restoring the equilibrium between oxidants and anti-oxidants, thus mitigating renal oxidative stress. Furthermore, in central and peripheral nervous system disorders, *N. sativa* and TQ modulated the pro-oxidant-anti-oxidant balance. These disorders are associated with oxidative damage to the nervous system, which can have profound implications for neuronal function and overall neurological health. However, through their anti-oxidant properties, *N. sativa* and TQ play a crucial role in maintaining the delicate equilibrium between pro-oxidants and anti-oxidants, thereby conferring protective and therapeutic effects in these disorders. Moreover, in diabetes-induced oxidative damage and hyperlipidemia, *N. sativa* and TQ have shown protective and therapeutic effects by modulating the oxidative/anti-oxidant pathways. Diabetes and hyperlipidemia are characterized by increased oxidative stress, which contributes to tissue damage and the development of diabetic complications. *N. sativa* and TQ, by their anti-oxidant properties, help restore the balance between oxidants and anti-oxidants, thereby alleviating oxidative damage associated with diabetes and hyperlipidemia. In addition, the plant and TQ have been found to possess therapeutic effects against oxidative damage in testicular injuries. The testes and ovaries are particularly vulnerable to oxidative damage due to their high metabolic activity and exposure to various toxic agents. Administration of *N. sativa* and TQ has been shown to mitigate oxidative damage in these reproductive organs, thereby highlighting their potential as therapeutic agents in the management of testicular and ovarian disorders.

Clinical studies also reported the anti-oxidant activity of the plant and TQ in disorders associated with oxidative stress, leading to improvement of these disorders by reducing oxidant but enhancing anti-oxidant marker levels.

Finally, the possible molecular mechanisms underlying the anti-oxidant effects of *N. sativa* and TQ have been elucidated in Figure 4. This figure provides a visual representation of the intricate cellular pathways through which *N. sativa* and TQ exert their protective and therapeutic effects by modulating the oxidant/anti-oxidant balance. By comprehending these molecular mechanisms, researchers and clinicians can gain valuable insights into the potential therapeutic applications of *N. sativa* and TQ in the management of oxidative stress-related disorders.

## Conclusion

Oxidant/anti-oxidant balance improvement is discussed as a therapeutic mechanism of *N. sativa* and TQ. Indeed, oxidant/anti-oxidant pathways have been shown as beneficial effects of *N. sativa* and TQ in oxidative stress-associated disorders along with other cellular signaling pathways. The efficacy of *N. sativa* and TQ in various disorders (such as respiratory, cardiovascular, reproductive system, renal, gastrointestinal, diabetes mellitus, cardiotoxicity, nephrotoxicity, gastrointestinal toxicity, neurotoxicity, testicular toxicity, and I/R injury), revealed its potential use with other treatment programs. Therefore, after careful pharmacological examination, *N. Sativa* and TQ may be used for medicinal purposes as anti-oxidant agents in oxidative stress-associated disorders. 

## Authors’ Contributions

MR A collected data; MR A and S S discussed the results and prepared a draft manuscript; MH B supervised, directed, and managed the study; MR A, S S, and MH B approved the final version to be published.

## Funding

This research did not receive any specific grant from funding agencies in the public, commercial, or not-for-profit sectors.

## Conflicts of Interest

The authors declare no conflicts of interest.
